# Synthesis, Characterization and Activity Evaluation of Matrinic Acid Derivatives as Potential Antiproliferative Agents

**DOI:** 10.3390/molecules18055420

**Published:** 2013-05-10

**Authors:** Fan Chao, Dong-En Wang, Rui Liu, Qin Tu, Jian-Jun Liu, Jinyi Wang

**Affiliations:** 1 College of Science, Northwest A&F University, Yangling 712100, China; E-Mail: chaofan@nwsuaf.edu.cn (F.C.); 2 College of Forestry, Northwest A&F University, Yangling 712100, China

**Keywords:** matrinic acid derivative, synthesis, antiproliferative activity

## Abstract

A series of new matrinic acid derivatives **5a**–**e** was synthesized. The chemical structures of the synthesized compounds were confirmed by ^1^H-NMR, ^13^C-NMR, and electrospray ionization mass spectroscopy. The anti-tumor activities were also investigated *in vitro* by evaluating the effect of synthesized compounds on the proliferation of A375, A549, HeLa, and HepG2 cells. Compound **5e** was found to be the most potent against A375 and HeLa cells, with IC_50_ values of 37 and 75.5 μg/mL, respectively. Compounds **5b**, **5c**, **5g**, and **5h** also exhibited antiproliferative activities against A549 cells, with IC_50_ values within the 36.2–47 μg/mL range. For HepG2 cells, **5e** and **5i**, with IC_50_ values of 78.9 and 61 μg/mL, respectively, showed higher antiproliferative activity than taxol.

## 1. Introduction

**Figure 1:**
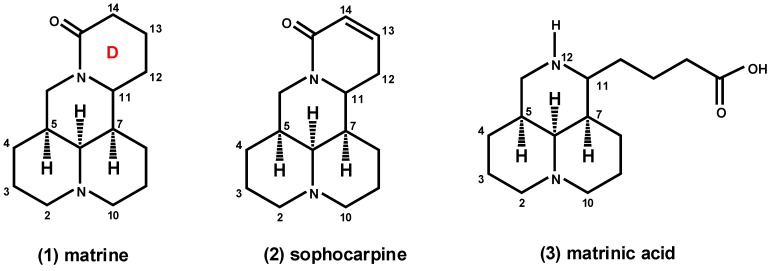
Chemical structures of matrine (**1**), sophocarpine (**2**), and matrinic acid (**3**).

Matrine (Figure 1) is one of the main alkaloid components that can be isolated from *Sophora flavescens* (Kushen), *Subprostrata* (Shandougen), and *Alopecuroides* (Kudouzi) [1,2,3]. Matrine possesses a broad spectrum of biological activities, such as antipyretic [4], anti-inflammatory [5], antimicrobial [6], antiviral [7], immunoinhibitory [8], antifibrotic [9], analgesic [10], antiarrhythmic [11], and anti-diarrheal [12] effects. In China, matrine is widely used as an effective drug for the treatment of hepatitis, colpitis, and chronic cervicitis [13]. Recently, the anti-tumor activity of matrine has gained interest [14,15,16,17,18,19,20], and several studies have demonstrated the antitumor effects of matrine by inhibiting the proliferation and inducing apoptosis of certain cancer cells, such as human hepatoma cancer [15], lung cancer [15], gastric cancer [16,17], leukemia K-562 [18], and glioma [19] cells. Matrine can also inhibit the invasiveness and metastasis of the human malignant melanoma cell line A375 [20] and HeLa cells [21]. However, the moderate anti-tumor activities [14] of matrine limit its use as a drug for clinical applications, and consequently, development of matrine derivatives is necessary to discover more effective drug candidates.

Given the structural stability of matrine, previous studies have mainly focused on the modification of sophocarpine (**2**, Figure 1), whose structure closely resembles that of matrine. Recently, some matrine derivatives comprising newly introduced substituents to the D-ring at the C-13 or C-14 positions have shown increased anti-hepatitis B virus (HBV) [22], anti-inflammatory [23], and anticancer [14,24] activities. Moreover, some researchers have opened the D-ring of matrine to generate matrinic acid (**3**, Figure 1), which is easier to modify. Wang *et al.* [13] demonstrated that the amide bond may be necessary for the anti-tumor activities of matrine, since when the D-ring is opened and the amide bond is broken, the antiproliferative activities are lost. Jiang *et al.* [25] synthesized N-substituted matrinic acid analogs and found that the D-ring of matrine may not be required for anti-HBV activity, whereas the carboxyl group is necessary for this activity. Introducing a substituent to the nitrogen atom at the 12-position also significantly improves the anti-HBV activity. Based on these results, we hypothesized that the substituents on the nitrogen atom at the 12-position and on the carboxyl group at the 11-position may play key roles in the anti-tumor activities of martrinic acid analogs. However, research on the activities after modification at the 12-positon or of the carboxyl group of these matrinic acid derivatives is limited.

In the present work, we modified matrinic acid at the 12-position and on the carboxyl group. We initially introduced benzyl as a substituent to the nitrogen atom at the 12-position because benzyl may significantly increase the activities of matrine [25]. We then shifted our interest to the modification of the carboxyl group. We converted the carboxyl group to its amide and ester derivatives. Thus, a series of new matrinic acid derivatives was synthesized. The antiproliferative activities of these derivatives were investigated by 3-(4,5-dimethylthiazol-2-diphenyltetrazolium) bromide (MTT) assay against human malignant melanoma (A375), human lung adenocarcinoma (A549), human cervical carcinoma (HeLa) and human hepatocellular carcinoma (HepG2) cells.

## 2. Results and Discussion

### 2.1. Chemistry

**Scheme 1:**
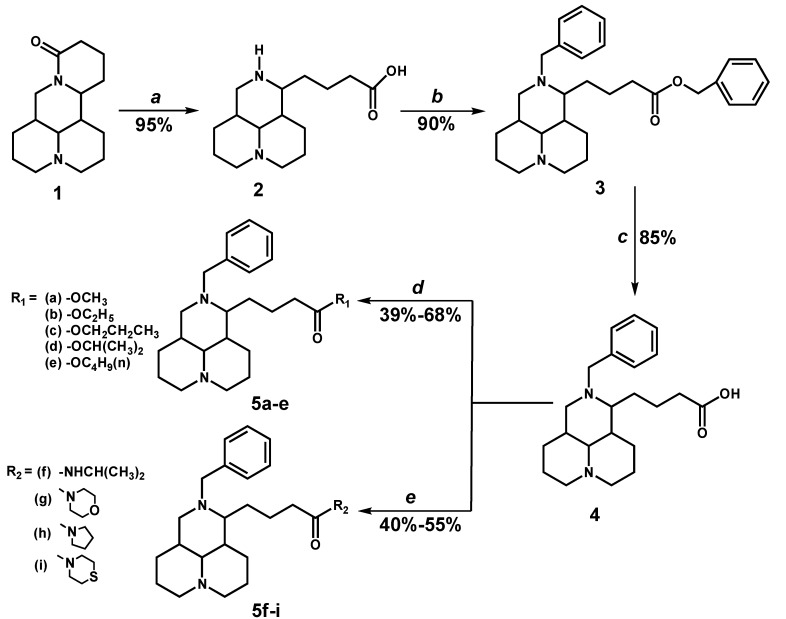
Synthesis of matrinic acid derivatives.

Nine new matrinic acid derivatives, **5a**–**i**, were synthesized in a four–step reaction using commercially available **1** as a starting material (Scheme 1). The desired matrinic acid (**2**) was prepared in a yield of 95% by the hydrolysis of **1** in 15% aqueous NaOH. Product **3** was generated with a satisfactory yield of 90% through the reaction of benzyl chloride with **2** in the presence of potassium carbonate [24,25]. The key intermediate **4** was obtained by the hydrolysis of **3** using sodium hydroxide as the base catalyst [25] and then converted into **5a**–**e** through esterification reactions with a series of alcohols (*i.e.*, methanol, ethanol, propanol, isopropanol, and *n*-butanol) in the presence of H_2_SO_4_. The yields ranged from 40% to 68.5%. Compounds **5f**–**i** were prepared in yields ranging from 40% to 56 % by the amidation reactions of **4** with isopropylamine, pyrrolidine, morpholine, or thiomorpholine, using 4-dimethylaminopyridine (DMAP) as a catalyst and 1-ethyl-(3-dimethylaminopropyl)-carbodiimide hydrochloride (EDC) as a dessicant. The structures of all synthesized compounds were confirmed by mass spectrometry, ^1^H- and ^13^C-NMR spectroscopy. 

*Reagent and conditions*: (**a**) 15% aqueous NaOH, 100 °C, 10 h; (**b**) ClCH_2_Ph, K_2_CO_3_, CH_3_CN, 80 °C, 12 h; (**c**) NaOH, CH_3_CH_2_OH, 80 °C, 4 h; (**d**) R_1_-H, H_2_SO_4_, reflux, 6 h; (**e**) R_2_-H, EDC/DMAP, CH_2_Cl_2_, 25 °C, 24 h.

### 2.2. Antiproliferative Activity

**Figure 2:**
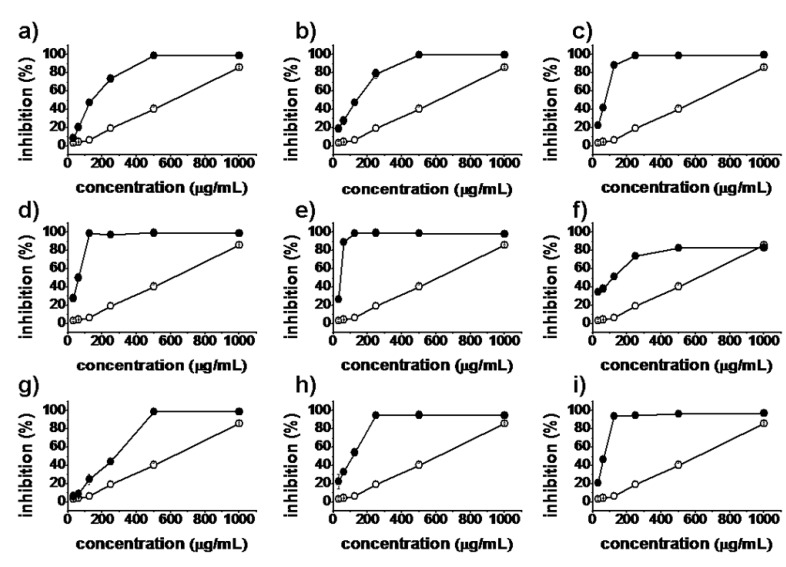
Inhibition percentages of **5a**–**i** (**a**–**i**, respectively) against A375 cells at concentrations of 31.25, 62.5, 125, 250, 500, and 1,000 μg/mL. Matrine (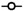), synthesized compounds (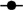). Results are expressed as the mean ± SD of data obtained from three independent experiments.

**Figure 3:**
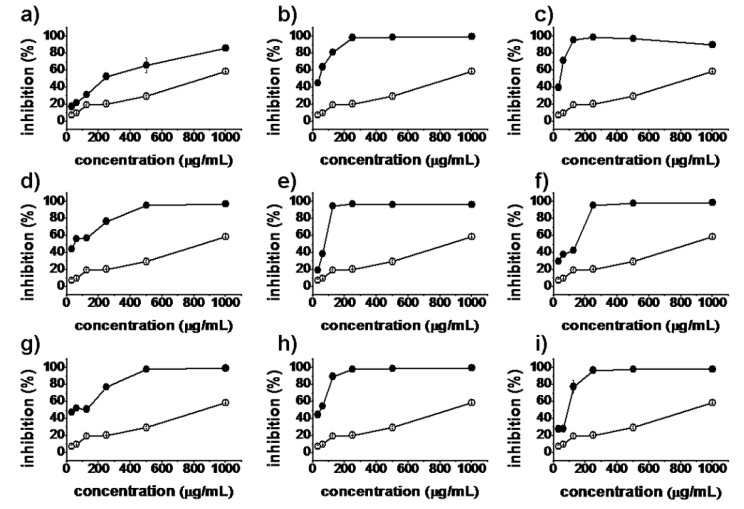
Inhibition percentages of **5a**–**i** (**a**–**i**, respectively) against A549 cells at concentrations of 31.25, 62.5, 125, 250, 500, and 1,000 μg/mL. Matrine (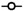), synthesized compounds (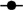). Results are expressed as the mean ± SD of data obtained from three independent experiments.

**Figure 4:**
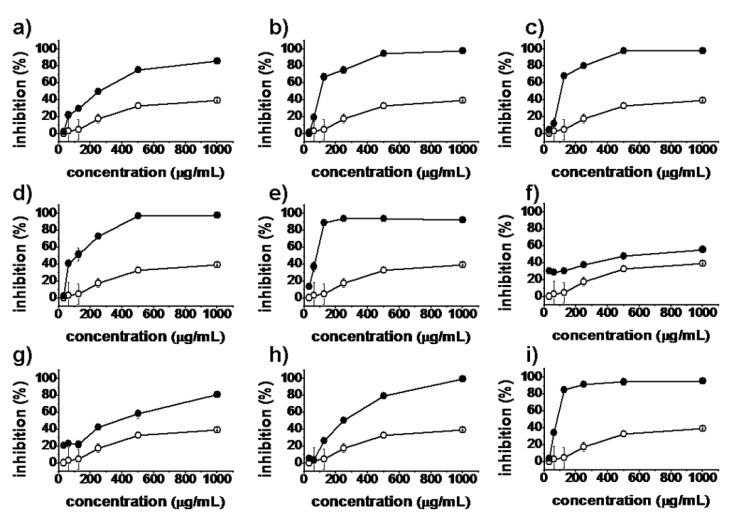
Inhibition percentages of **5a**–**i** (**a**–**i**, respectively) against HeLa cells (**3**) at concentrations of 31.25, 62.5, 125, 250, 500, and 1,000 μg/mL. Matrine (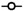), synthesized compounds (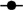). Results are expressed as the mean ± SD of data obtained from three independent experiments.

**Figure 5:**
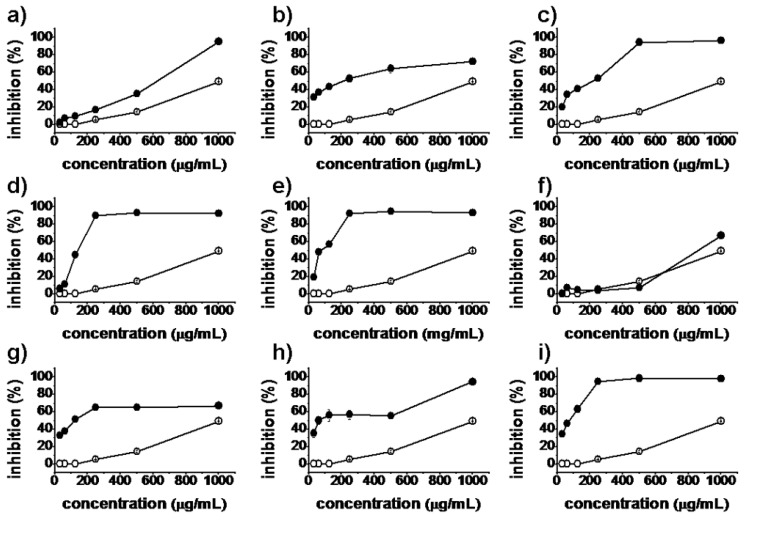
Inhibition percentages of **5a**–**i** (**a**–**i**, respectively) against HepG2 cells (**4**) at concentrations of 31.25, 62.5, 125, 250, 500, and 1,000 μg/mL. Matrine (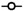), synthesized compounds (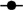). Results are expressed as the mean ± SD of data obtained from three independent experiments.

*In vitro* antiproliferative activities of all synthesized matrinic acid derivatives were evaluated by the MTT assay against the human cancer cell lines A375, A549, HeLa and HepG2. Matrine and taxol were used as positive controls. As shown in Figure 2, Figure 3, Figure 4 and Figure 5, the prepared matrinic acid derivatives **5a**–**i** exhibited different antiproliferative activities, depending on the changes in their chemical structures. High doses of these compounds also showed more potent antiproliferative activities. Among the tested compounds, **5e** and **5i** displayed the most effective antiproliferative activities against the four cancer cell lines, with inhibition rates above 90% at the tested concentration of 250 μg/mL. Compound **5f** showed a low inhibition activity against HepG2 cells similar to that of matrine. Furthermore, the antiproliferative activities of **5a**–**i** against the four cancer cell lines were much stronger than that of matrine.

Table 1 shows the lipophilicity and half-maximal inhibitory concentration (IC_50_) values of all synthesized compounds against A375, A549, HeLa, and HepG2 cells. Results showed that consistent with a previous report [13], matrinic acid **2** and the key intermediate **4** lost almost all their antiproliferative activities, possibly because opening the D-ring of matrine broke the amide bond. Thus, we first converted the carboxyl group of **4** to its amide derivatives. As expected, the resulting compounds **5f**–**i** exhibited stronger antiproliferative activities against the four cancer cell lines (except for **5f** on HepG2 cells), with IC_50_ values 2-fold to 20-fold higher than that of matrine. In addition, introduction of cyclic amine substituents may greatly improve the observed antiproliferative activities of said derivatives compared with a noncyclic amine substituent. The data showed that **5g**, **5h**, and **5i** were more active than **5f**. Among all these compounds, **5i** showed highest inhibition efficiency against the four cancer cell lines, with IC_50_ values reaching 61.0 μg/mL against HepG2 cells (even higher than that of taxol).

Next, the carboxyl group of **4** was converted to its ester derivatives. Surprisingly, the products **5a**–**e** had significantly higher antiproliferative activities against the four cancer cell lines than the parent matrine, suggesting that the amide bond was not the only requirement for anticancer activity. Another interesting observation was that increased length of the alkyl portion of the alcohols used to form the esters enhanced the antiproliferative activities of the matrinic acid derivatives. For example, **5a**, **5b**, **5c**, and **5e** exhibited gradually increasing inhibition activities against A375, HeLa, and HepG2 cells. Moreover, the antiproliferative activities of **5c** and **5d** against A375, HeLa, and HepG2 cells were almost similar. Considering the structural features of **5a**–**e**, we deduced that the possible reason for these results was that the alcohols used to form the esters changed the lipophilicity of these derivatives, which in turn influenced their interaction with tumor cells [26]. With increased length of the alkyl portion of the alcohols, the lipophilicity of the matrinic acid derivatives were enhanced and the interactions with tumor cells improved. The increased lipophilicity of synthesized compounds may enhance their permeability into cell membrane and then influence the bioactivity. Compound **5c** had relatively close lipophilicity to **5d**, resulting in similar antiproliferative activities against A375, HeLa, and HepG2 cells. This explanation can also be suitable for **5f**, which was derived using isopropylamine instead of isopropanol. The antiproliferative activity of **5f** was significantly lower than that of **5d** possibly because of the weaker lipophilicity of isopropylamine.

**Table 1:** Lipophilicity and IC_50_ of matrine, taxol, and synthesized matrinic acid derivatives against A375, A549, HeLa, and HepG2 cells.

Compound	*C*log*P ^a^*	IC_50_ (μg/mL)			
		A375	A549	HeLa	HepG2
**Matrine**	1.5	551.1 ± 22	1022.0 ± 16.5	1238.0 ± 5.7	1156.5 ± 11.9
**2**	−0.704	1345.7 ± 125.6	NA *^b^*	NA *^b^*	NA *^b^*
**4**	1.572	853.1 ± 4.2	NA *^b^*	2201.5 ± 3.5	734.9 ± 24.4
**5a**	4.218	138.7 ± 12.8	235.5 ± 1.6	223.5 ± 4.9	447.9 ± 34.4
**5b**	4.747	116.7 ± 17	37.9 ± 2.3	121.2 ± 4.8	176.3 ± 5.6
**5c**	5.276	60.7 ± 1.2	36.2 ± 1.3	118.8 ± 9.3	128.4 ± 2.3
**5d**	5.056	58.5 ± 0.1	45.9 ± 0.1	102.9 ± 1.4	138.9 ± 9.2
**5e**	5.805	37.0 ± 0.2	64.3 ± 6.2	75.5 ± 5.0	78.9 ± 0.4
**5f**	3.796	82.8 ± 17.2	80.8 ± 2.7	758.2 ± 16.4	1383.3 ± 72.2
**5g**	3.233	252 ± 1.4	47.0 ± 8.2	297.0 ± 27.6	143.6 ± 8.2
**5h**	3.458	70.8 ± 7.0	41.5 ± 1.5	223.5 ± 4.9	100.9 ± 6.9
**5i**	3.958	59.3 ± 0.9	70.6 ± 8.6	89.2 ± 0.6	61.0 ± 1.4
**Taxol**	—	31.3 ± 1.0	39.4 ± 0.9	18.3 ± 3.1	85.1 ± 6.2

*^a^* Lipophilicity of the synthesized compounds and matrine is expressed in the term of their ClogP values which were calculated by the software ChemDraw Ultra 12.0; *^b^* NA: no activity.

## 3. Experimental 

### 3.1. General

Matrine (>98%) was purchased from Xi'an Natural Field Bio-Technique Co., Ltd. (Xi’an, China). Taxol was purchased from Xi’an Haoxuan Biology Technology Co., Ltd. (Xi’an, China). Trypsin and MTT were obtained from Amersco Inc. (Solon, OH, USA). Peniclllin and streptomycin were from Sigma-Aldrich (St. Louis, MO, USA). Dulbecco’s modified eagle medium (DMEM), and fetal bovine serum (FBS) were from Gibco Invitrogen Corporation (Carlsbad, CA, USA). Secondary amines were from Aladdin (Shanghai, China). Dichloromethane was purified by distillation over CaH_2_ prior to use. All other reagents and solvents were analytical grade; they were supplied by local commercial suppliers and used without further purification unless otherwise noted. All reactions were carried out in oven-dried flasks with stirring. Column chromatography was conducted using silica gel 60 (Qingdao Haiyang Chem. Co., Ltd., Shandong, China). Reactions were monitored by thin-layer chromatography (TLC) using silica gel 60 GF254 (Qingdao Haiyang Chem. Co., Ltd., Shandong, China) and visualized under UV (254 nm) light (ZF-6 type-III ultraviolet analyzer; Shanghai Jiapeng Technology Co., Ltd., Shanghai, China). Melting points were determined using a digital melting point apparatus (Zhengzhou Mingze Technology Co., Ltd., Zhengzhou, China) and were uncorrected. ^1^H-NMR and ^13^C-NMR spectra were recorded on a Bruker Avance DMX 500 MHz/125 MHz spectrometer or Bruker Avance DMX 400 MHz/100 MHz spectrometer (Bruker, Billerica, MA, USA), with chemical shifts reported in parts per million in CDCl_3_, CD_3_OD or D_2_O (tetramethylsilane as an internal standard); *J* values were given in Hertz. Proton coupling patterns were described as singlet (s), doublet (d), triplet (t), and multiplet (m). Electrospray ionization mass spectroscopy (ESI-MS) data were obtained using a Thermo Scientific LCQ FLEET mass spectrometer equipped with an electrospray ion source and controlled by Xcalibur software (Thermo Fisher Scientific, Waltham, MA, USA).

### 3.2. General Procedure for the Preparation of Matrinic Acid ***2***

Matrine (**1**, 2.44 g, 10 mmol) was added to 15% aqueous NaOH solution (80 mL) and the mixture was stirred at 100 °C for 10 h and then cooled to room temperature, neutralized with 20% H_2_SO_4_ solution in an ice bath, and concentrated *in vacuo*. The residue was dissolved in CH_3_OH in a hot-water bath and filtered. The filtrate was concentrated to 5 mL and then acetone (40 mL) was added with vigorous stirring to obtain a white precipitate, which was isolated by filtration and dried *in vacuo* to yield **2** as a white solid (2.53 g, 95%), m.p. 186–187 °C. ^1^H-NMR (500 MHz, D_2_OD and CD_3_OD) *δ* 3.52 (s, 1H), 3.45–3.37 (m, 2H), 3.04 (d, *J* = 10.2 Hz, 1H), 2.93–2.79 (m, 2H), 2.34 (s, 1H), 2.32–2.27 (m, 1H), 2.27–2.18 (m, 1H), 2.10 (d, *J* = 9.6 Hz, 3H), 1.99 (d, *J* = 12.5 Hz, 1H), 1.93–1.80 (m, 2H), 1.80–1.66 (m, 5H), 1.65–1.48 (m, 5H). ^13^C-NMR (125 MHz, D_2_OD and CD_3_OD) *δ* 180.96 (COOH), 61.91, 56.53, 56.44, 52.23, 43.38, 38.29, 36.71, 33.09, 30.11, 26.36, 25.32, 20.19, 20.10, 19.80. ESI-MS *m*/*z*: 267.27 (M+H)^+^.

### 3.3. General Procedure for the Preparation of ***3***

Benzyl chloride (2 mL) was added to a solution of matrinic acid **2** (2.53 g, 9.5 mmol) and K_2_CO_3_ (6.56 g, 47.5 mmol) in CH_3_CN (50 mL). The mixture was stirred and refluxed at 70 °C for 12 h. The solution was filtered to remove solids, and the filtrate was concentrated *in vacuo*. The resulting residual was dissolved in ethyl acetate (50 mL), and washed with water and saturated NaCl. The organic layer was dried with MgSO_4_ and concentrated *in vacuo* to give **3** as a light yellow oil (3.81 g, 90%). ^1^H-NMR (500 MHz, CDCl3) *δ* 7.32 (d, *J* = 6.7 Hz, 6H), 7.29 (d, *J* = 7.5 Hz, 2H), 7.26 (d, *J* = 5.4 Hz, 1H), 7.21 (t, *J* = 7.1 Hz, 1H), 5.19–4.97 (m, 2H, OCH_2_), 4.06 (d, *J* = 13.3 Hz, 1H), 3.08 (d, *J* = 13.3 Hz, 1H), 2.84 (t, *J* = 9.5 Hz, 2H), 2.76 (d, *J* = 11.0 Hz, 1H), 2.60 (t, *J* = 11.8 Hz, 1H), 2.43–2.22 (m, 3H), 2.03 (s, 1H), 1.96–1.83 (m, 3H), 1.80 (d, *J* = 7.2 Hz, 2H), 1.75–1.54 (m, 6H), 1.47–1.29 (m, 5H). ^13^C-NMR (125 MHz, CDCl_3_) *δ* 173.54, 140.57, 136.07, 128.70, 128.55, 128.27, 128.21, 128.19, 126.54, 66.12 (OCH_2_), 64.61, 57.65 (N-CH_2_-Ph), 57.48, 57.42, 56.79, 52.28, 38.09, 34.59, 34.08, 28.58, 28.21, 27.34, 21.67, 21.44, 19.27. ESI-MS *m*/*z*: 447.37 (M+H)^+^.

### 3.4. General Procedure for the Preparation of ***4***

Compound **3** (3.81 g, 8.5 mmol) was added to a solution of NaOH (2 g, 50 mmol) in ethanol (40 mL). The mixture was stirred and refluxed at 80 °C for 4 h. After cooling to room temperature, the solution was neutralized with 20% H_2_SO_4_ solution and concentrated *in vacuo*. The residue was dissolved in water and thoroughly washed with ethyl acetate. The water layer was concentrated and dried *in vacuo*. CH_3_OH was added to the resulting solid, and the mixture was refluxed for 20 min. The hot solution was immediately filtered, and the filtrate was concentrated *in vacuo* to give **4** as a yellow solid (2.59 g, 85%), m.p. 95–96 °C. ^1^H-NMR (400 MHz, CDCl_3_) *δ* 10.03 (s, 1H, COOH), 7.41 (d, *J* = 5.9 Hz, 2H), 7.34–7.23 (m, 3H), 4.36 (d, *J* = 13.2 Hz, 1H), 3.70 (d, *J* = 13.2 Hz, 1H), 3.30 (d, *J* = 10.9 Hz, 1H), 3.07–2.77 (m, 3H), 2.56 (dd, *J* = 12.1, 3.5 Hz, 1H), 2.41 (d, *J* = 15.5 Hz, 1H), 2.29 (d, *J* = 15.8 Hz, 1H), 2.25–2.12 (m, 2H), 2.03 (d, *J* = 2.2 Hz, 1H), 1.98 (d, *J* = 9.9 Hz, 1H), 1.88 (dd, *J* = 33.2, 8.6 Hz, 5H), 1.71–1.54 (m, 2H), 1.51–1.31 (m, 5H), 1.25 (s, 1H). ^13^C-NMR (100 MHz, CDCl3) *δ* 178.22 (COOH), 134.19, 129.97(2C), 128.75(2C), 128.14, 63.17, 58.01, 56.77, 56.59, 54.50, 50.10, 36.85, 36.06, 31.67, 28.86, 27.17, 26.54, 20.95, 20.58, 20.40. ESI-MS *m*/*z*: 357.31 (M+H)^+^.

### 3.5. General Procedure for the Preparation of ***5a**–**e***

A drop of H_2_SO_4_ was added to a solution of **4** (200 mg, 0.56 mmol) in methanol (20 mL). The mixture was stirred and refluxed at 80 °C for 4 h. After cooling, 20% aqueous NaOH was added to the solution to neutralize the acid. The reaction mixture was poured into saturated aqueous NaHCO_3_ (50 mL) and extracted with EtOAc (3 × 70 mL). The organic extracts were dried over Na_2_SO_4_ and concentrated *in vacuo* to give a yellow oil, which was purified by chromatography (silica, 20:1 CH_2_Cl_2_:CH_3_OH) to yield **5a** as a light yellow solid. Compounds **5b**–**e** were prepared using similar methods, replacing methanol with the appropriate alcohol, and their structures were confirmed by ^1^H-NMR, ^13^C-NMR, and ESI-MS. The data are as follows:

*12-Benzylmatrinic acid methyl ester* (**5a**): light yellow solid, m.p. 72–73 °C, yield 40%.^1^H-NMR (400 MHz, CDCl_3_) *δ* 7.35 (t, *J* = 7.0 Hz, 2H), 7.30 (d, *J* = 10.5 Hz, 2H), 7.23 (t, *J* = 7.0 Hz, 1H), 4.11 (d, *J* = 13.3 Hz, 1H), 3.65 (s, 3H, OCH_3_), 3.12 (d, *J* = 13.3 Hz, 1H), 2.93–2.74 (m, 3H), 2.63 (t, *J* = 11.9 Hz, 1H), 2.44–2.25 (m, 3H), 2.07 (s, 1H), 2.01–1.89 (m, 3H), 1.84–1.54 (m, 8H), 1.52–1.30 (m, 5H). ^13^C-NMR (100 MHz, CDCl_3_) *δ* 174.16, 140.44, 128.70 (2C), 128.19 (2C), 126.55, 64.57, 57.62, 57.49, 57.38, 56.81, 52.23, 51.45 (OCH_3_), 38.10, 34.29, 34.07, 28.60, 28.17, 27.30, 21.62, 21.40, 19.22. ESI-MS *m/z*: 371.31 (M+H)^+^.

*12-Benzylmatrinic acid ethyl ester* (**5b**): yellow solid, m.p. 97–98 °C, yield 55.3%. ^1^H-NMR (500 MHz, CDCl_3_) *δ* 7.39–7.31 (m, 2H), 7.31–7.25 (m, 2H), 7.22 (s, 1H), 4.09 (d, *J* = 7.3 Hz, 3H, CH and OCH_2_), 3.09 (d, *J* = 12.6 Hz, 1H), 2.85 (s, 2H), 2.76 (d, *J* = 10.1 Hz, 1H), 2.60 (s, 1H), 2.34 (d, *J* = 8.9 Hz, 1H), 2.27 (s, 2H), 2.04 (s, 1H), 1.99–1.84 (m, 3H), 1.84–1.53 (m, 8H), 1.48–1.30 (m, 5H), 1.22 (t, *J* = 6.9 Hz, 3H, CH_3_). ^13^C-NMR (125 MHz, CDCl_3_) *δ* 173.69, 140.62, 128.67 (2C), 128.19 (2C), 126.52, 64.63, 60.22 (OCH_2_), 57.64, 57.51, 57.41, 56.77, 52.27, 38.18, 34.62, 34.12, 28.66, 28.21, 27.35, 21.66, 21.43, 19.33, 14.22 (CH_3_). ESI-MS *m/z*: 385.32 (M+H)^+^.

*12-Benzylmatrinic acid propyl ester* (**5c**): yellow solid, m.p. 80–81 °C, yield 65.3%. ^1^H-NMR (400 MHz, CDCl_3_) *δ* 7.35 (d, *J* = 7.1 Hz, 2H), 7.29 (t, *J* = 7.4 Hz, 2H), 7.21 (t, *J* = 7.1 Hz, 1H), 4.12 (d, *J* = 13.3 Hz, 1H), 3.99 (t, *J* = 6.7 Hz, 2H, OCH_2_), 3.14 (d, *J* = 13.3 Hz, 1H), 2.96–2.76 (m, 3H), 2.64 (t, *J* = 12.0 Hz, 1H), 2.37 (dd, *J* = 11.5, 4.0 Hz, 1H), 2.33–2.24 (m, 2H), 2.11 (s, 1H), 2.02–1.66 (m, 10H), 1.66–1.55 (m, 3H), 1.49–1.31 (m, 5H), 0.91 (t, *J* = 7.4 Hz, 3H). ^13^C-NMR (100 MHz, CDCl_3_) *δ* 173.76, 139.95, 128.75 (2C), 128.22 (2C), 126.66, 65.87 (OCH_2_), 64.52, 57.51 (2C), 57.29, 56.74, 52.05, 38.00, 34.48, 33.90, 28.56, 27.99, 27.16, 21.97 (CH_2_-CH_3_), 21.44, 21.23, 19.28, 10.41 (CH_3_). ESI-MS *m/z*: 399.36 (M+H)^+^.

*12-Benzylmatrinic acid isopropyl ester* (**5d**): light yellow solid, m.p. 87–88 °C, yield 59.5%. ^1^H-NMR (500 MHz, CDCl_3_) *δ* 7.34 (d, *J* = 7.3 Hz, 2H), 7.29 (t, *J* = 7.5 Hz, 2H), 7.21 (t, *J* = 7.2 Hz, 1H), 4.98 (hept, *J* = 6.2 Hz, 1H, OCH), 4.10 (d, *J* = 13.3 Hz, 1H), 3.10 (d, *J* = 13.2 Hz, 1H), 2.85 (s, 2H), 2.77 (d, *J* = 10.8 Hz, 1H), 2.61 (t, *J* = 11.8 Hz, 1H), 2.34 (dd, *J* = 11.3, 3.1 Hz, 1H), 2.30–2.18 (m, 2H), 2.04 (s, 1H), 1.98–1.65 (m, 10H), 1.58 (t, *J* = 10.2 Hz, 1H), 1.45–1.30 (m, 5H), 1.19 (d, *J* = 6.3 Hz, 6H, (CH_3_)_2_). ^13^C-NMR (125 MHz, CDCl_3_) *δ* 173.21, 140.55, 128.70 (2C), 128.17 (2C), 126.53, 67.40 (OCH), 64.62, 57.63, 57.52, 57.40, 56.74, 52.23, 38.18, 34.96, 34.06, 28.69, 28.20, 27.34, 21.84 (2×CH_3_), 21.65, 21.41, 19.43. ESI-MS *m/z*: 399.34 (M+H)^+^.

*12-Benzylmatrinic acid butyl ester* (**5e**): yellow solid, m.p. 90–91 °C, yield 68.5%. ^1^H-NMR (500 MHz, CDCl_3_) *δ* 7.33 (d, *J* = 7.2 Hz, 2H), 7.28 (t, *J* = 7.5 Hz, 2H), 7.20 (t, *J* = 7.2 Hz, 1H), 4.09 (d, *J* = 13.4 Hz, 1H), 4.06–4.00 (m, 2H, OCH_2_), 3.10 (d, *J* = 13.4 Hz, 1H), 2.91–2.80 (m, 2H), 2.77 (d, *J* = 11.3 Hz, 1H), 2.62 (t, *J* = 11.9 Hz, 1H), 2.34 (dd, *J* = 11.5, 4.0 Hz, 1H), 2.26 (dd, *J* = 12.3, 6.8 Hz, 2H), 2.04 (s, 1H), 1.98–1.64 (m, 10H), 1.63–1.52 (m, 3H), 1.44–1.29 (m, 7H), 0.90 (t, *J* = 7.4 Hz, 3H, CH_3_). ^13^C-NMR (125 MHz, CDCl_3_) *δ* 173.73, 140.21, 128.70 (2C), 128.16 (2C), 126.56, 64.51, 64.11 (OCH_2_), 57.55, 57.48, 57.31, 56.70, 52.11, 38.04, 34.52, 33.94, 30.65 (CH_2_-CH_2_-CH_3_), 28.60, 28.10, 27.24, 21.53, 21.31, 19.32, 19.12 (CH_2_-CH_3_), 13.71(CH_3_). ESI-MS *m/z*: 413.37 (M+H)^+^.

### 3.6. General Procedure for the Preparation of ***5f**–**i***

Compound **4** (200 mg, 0.56 mmol) was dissolved in dry CH_2_Cl_2_ (10 mL), and a solution of EDC•HCl (161.1 mg, 0.84 mmol) in CH_2_Cl_2_ (10 mL), was added dropwise at 0 °C. The mixture was stirred for 30 min at room temperature. Then, DMAP and 0.48 mL (5.6 mmol) of isopropylamine were sequentially added. The reaction solution was stirred at room temperature until TLC indicated that conversion was complete (24 h). The mixture was washed with water and brine. After drying over Na_2_SO_4_, the organic layer was concentrated under reduced pressure, and the residue was purified by chromatography (silica, 15:1 CH_2_Cl_2_–CH_3_OH) to give **5f** as a yellow solid. The desired products **5g**–**i** were prepared using similar methods using the corresponding amines, and their structures were confirmed by ^1^H-NMR, ^13^C-NMR, and ESI-MS. The data are listed as follows.

*12-Benzylmatrinic acid isopropyl amide* (**5f**): yellow solid, m.p. 97–98 °C, yield 40%. ^1^H-NMR (400 MHz, CDCl_3_) *δ* 7.35 (d, *J* = 7.0 Hz, 2H), 7.32–7.27 (m, 2H), 7.22 (t, *J* = 7.1 Hz, 1H), 5.40 (s, 1H, NH), 4.16–3.87 (m, 2H), 3.17 (d, *J* = 13.6 Hz, 1H), 2.99–2.75 (m,3H), 2.69 (t, *J* = 11.6 Hz, 1H), 2.37 (dd, *J* = 11.5, 3.9 Hz, 1H), 2.13–2.00 (m, 2H), 1.99–1.81 (m, 4H), 1.70 (d, *J* = 6.7 Hz, 6H), 1.61–1.53 (m, 1H), 1.46–1.31 (m, 5H), 1.25 (s, 1H), 1.10 (dd, *J* = 6.6, 2.9 Hz, 6H, (CH_3_)_2_). ^13^C-NMR (100 MHz, CDCl_3_) *δ* 172.10, 140.47, 128.65 (2C), 128.23 (2C), 126.57, 64.58, 57.56, 57.41, 57.34, 56.55, 52.38, 41.18 (CH-CON), 37.87, 37.01, 33.80, 28.60, 28.09, 27.22, 22.79 (2×CH_3_), 21.55, 21.32, 20.12. ESI-MS *m/z*: 398.39 (M+H)^+^.

*12-Benzyl-matrinic acid morpholine amide* (**5g**): yellow solid, m.p. 84–85 °C, yield 45.3%. ^1^H-NMR (400 MHz, CDCl_3_) *δ* 7.35 (d, *J* = 7.4 Hz, 2H), 7.32–7.27 (m, 2H), 7.22 (t, *J* = 7.0 Hz, 1H), 4.11 (d, *J* = 13.6 Hz, 1H), 3.68–3.55 (m, 6H, CH_2_-O-CH_2_ and NCH_2_), 3.37 (s, 2H, NCH_2_), 3.17 (d, *J* = 13.6 Hz, 1H), 3.01–2.74 (m, 3H), 2.67 (t, *J* = 11.9 Hz, 1H), 2.38 (d, *J* = 9.2 Hz, 1H), 2.32–2.17 (m, 2H), 2.09 (s, 1H), 1.99–1.62 (m, 11H), 1.52–1.30 (m, 5H). ^13^C-NMR (100 MHz, CDCl_3_) *δ* 171.65, 140.26, 128.57 (2C), 128.24 (2C), 126.62, 66.90 (OCH_2_), 66.60 (OCH_2_), 64.51, 57.62, 57.55, 57.32, 56.64, 52.29, 45.96 (CH_2_-CON), 41.81 (CH_2_-CON), 37.97, 33.89, 33.32, 28.76, 28.07, 27.23, 21.52, 21.33, 19.43. ESI-MS *m/z*: 426.43 (M+H)^+^.

*12-Benzylmatrinic acid pyrrolidine amide* (**5h**): yellow sild, m.p. 93–94 °C, yield 55.0%. ^1^H-NMR (400 MHz, CDCl_3_) *δ* 7.34 (d, *J* = 7.3 Hz, 2H), 7.29 (t, *J* = 5.7 Hz, 2H), 7.20 (t, *J* = 7.2 Hz, 1H), 4.12 (d, *J* = 13.5 Hz, 1H), 3.43 (t, *J* = 6.8 Hz, 2H, CH_2_-CON), 3.33 (t, *J* = 6.7 Hz, 2H, CH_2_-CON), 3.10 (d, *J* = 13.5 Hz, 1H), 2.84 (d, *J* = 7.0 Hz, 2H), 2.77 (d, *J* = 11.0 Hz, 1H), 2.60 (t, *J* = 11.9 Hz, 1H), 2.34 (dd, *J* = 11.4, 3.9 Hz, 1H), 2.28–2.13 (m, 2H), 2.04 (s, 1H), 2.00–1.85 (m, 5H), 1.84–1.65 (m, 10H), 1.47–1.30 (m, 5H). ^13^C-NMR (100 MHz, CDCl_3_) *δ* 171.59, 140.77, 128.61 (2C), 128.16 (2C), 126.44, 64.63, 57.69 (2C), 57.42, 56.95, 52.41, 46.56 (CH_2_-CON), 45.56 (CH_2_-CON), 38.28, 35.09, 34.23, 29.00, 28.23, 27.35, 26.12 (CH_2_-CH_2_-NOC), 24.40 (CH_2_-CH_2_-NOC), 21.69, 21.50, 19.09. ESI-MS *m*/*z*: 410.39 (M+H)^+^.

*12-Benzylmatrinic acid thiomorpholine amide* (**5i**): yellow oil, yield 56%. ^1^H-NMR (400 MHz, CDCl_3_) *δ* 7.38 (d, *J* = 7.3 Hz, 2H), 7.31 (t, *J* = 7.4 Hz, 2H), 7.24 (t, *J* = 7.1 Hz, 1H), 4.17 (s, 1H), 3.84 (dd, *J* = 10.4, 4.8 Hz, 2H, CH_2_-CON), 3.71–3.60 (m, 2H, CH_2_-CON), 3.26 (d, *J* = 12.0 Hz, 1H), 2.88 (dd, *J* = 69.5, 36.9 Hz, 4H), 2.60–2.54 (m, 4H), 2.43 (d, *J* = 9.7 Hz, 1H), 2.32–2.20 (m, 2H), 1.97 (d, *J* = 12.8 Hz, 5H), 1.83–1.63 (m, 6H), 1.52–1.34 (m, 5H), 1.29–1.21 (m, 1H). ^13^C-NMR (100 MHz, CDCl_3_) *δ* 171.27, 139.09, 128.85 (2C), 128.36 (2C), 126.94, 64.45, 57.41, 57.20, 56.70, 53.47, 52.03, 48.20 (CH_2_-CON), 44.15 (CH_2_-CON), 37.82, 33.60, 33.46, 29.72, 28.71, 28.12, 27.89 (CH_2_-S-), 27.40 (CH_2_-S), 21.32, 21.13, 19.51. ESI-MS *m/z*: 442.38 (M+H)^+^.

### 3.7. Antiproliferative Studies

The inhibitory effects of **5a**–**e** on HeLa, HepG2, A549, and A375 cell proliferation were evaluated *in vitro* by the MTT assay, according to a previously reported procedure [27], with slight modifications. HeLa and HepG2 cells were provided by the Cell Center of the Fourth Military Medical University (Xi’an, China). A549 and A375 cells were from the Chinese Academy of Sciences (Shanghai, China). The four selected cancer cell lines A375, A549, HeLa, and HepG2 were cultured using DMEM supplemented with 10% FBS, 100 units/mL penicillin, and 100 μg/mL streptomycin at 37 °C and 5% CO_2_ with 95% humidity. The cells were passaged at a ratio of 1:3 every 3 days to maintain them in the exponential growth phase. Prior to use, the cells were harvested by trypsinization with 0.25% trypsin in Ca^2+^- and Mg^2+^-free Hanks’ balanced salt solution at 37 °C. Trypsinization was stopped by adding fresh supplemented medium. The cell suspension was centrifuged at 1,000 rpm for 5 min. The cells were then resuspended in supplemented medium (1.0 × 10^6^ cells/well in six-well plates) for further use. Cells were seeded into 96-well microtiter plates (4.0 × 10^3^ cells per well) with 150 μL of fresh medium. After 24 h incubation, the tested compounds (150 μL, final concentrations of 31.25, 62.5, 125, 250, 500, or 1,000 μg/mL in the culture medium) were added to each well, and continuous culturing was performed for another 24 h. Afterwards, 5 mg/mL MTT (20 μL) was added to each well, which was then cultured for another 4 h under similar conditions. Finally, dimethylsulfoxide (150 μL) was added to terminate the reaction. The survival rate of the cancer cells was evaluated by measuring the optical density (*A*) on a microplate reader (model 680, BIO-RAD, Hercules, CA, USA) at 490 nm. All *in vitro* results were expressed as the cancer cell proliferation inhibition ratio according to the following formula:

[(*A*_control_ − *A*_test_)/*A*_control_] × 100%

where *A*_control_ and *A*_test_ are the optical densities of the control and test groups, respectively. All assays were done in triplicate. Experimental results are presented as the mean ± standard deviation (SD) of three parallel measurements. IC_50_ values were estimated using a non-linear regression algorithm (SPSS 19.0 Inc., Chicago, IL, USA).

## 4. Conclusions 

A series of new matrinic acid derivatives was synthesized by modifying matrinic acid (**2**) at the position 12 and the carboxyl group. The anti-tumor activity of the synthetized compounds was evaluating by assessing the compounds’ effect on A375, A549, HeLa and HepG2 cells proliferation. The results showed that the synthesized compounds exhibited relatively higher antiproliferative activities *in vitro* than the parent matrine (**1**). The results demonstrated that: (i) the amide bond on the carboxyl group may be required for anticancer activity, (ii) introduction of cyclic amine substituents to the carboxyl group can significantly enhance anticancer activity, (iii) conversion of the carboxyl group to its ester derivatives can also improve anticancer activity, and (iv) increasing the lipophilicity of matrinic acid esters can enhance anticancer activities against the four cancer cell lines tested. Among the derivatives, **5e** and **5i** exhibited the highest activities against the four cancer cell lines, and they were thus selected as potential anti-tumor drug candidates for further investigation. Our results also provided new information on the relationship between chemical structure and anti-tumor activity, which may help in exploring structural modifications of matrine and identifying other potential anti-tumor drug candidates.

## Supplementary Materials

^1^H and ^13^C-NMR spectra of the compounds **2**, **3**, **4**, and **5a-5i** are available in the supplementary materials. Supplementary materials can be accessed at: http://www.mdpi.com/1420-3049/18/5/5420/s1.
