# Supplementary material for: Synthesis, Characterization and Activity Evaluation of Matrinic Acid Derivatives as Potential Antiproliferative Agents
Source: Molecules. 2013 May 10;18(5):5420–33. doi: 10.3390/molecules18055420 (PMC6269743; doi:10.3390/molecules18055420)

Supplementary Materials

^{1}H and ^{13}C-NMR spectra of the compounds **2**, **3**, **4** and **5a–i**.

Figure S1. ^{1}H-NMR of **2** in D_2O and CD_3OD.

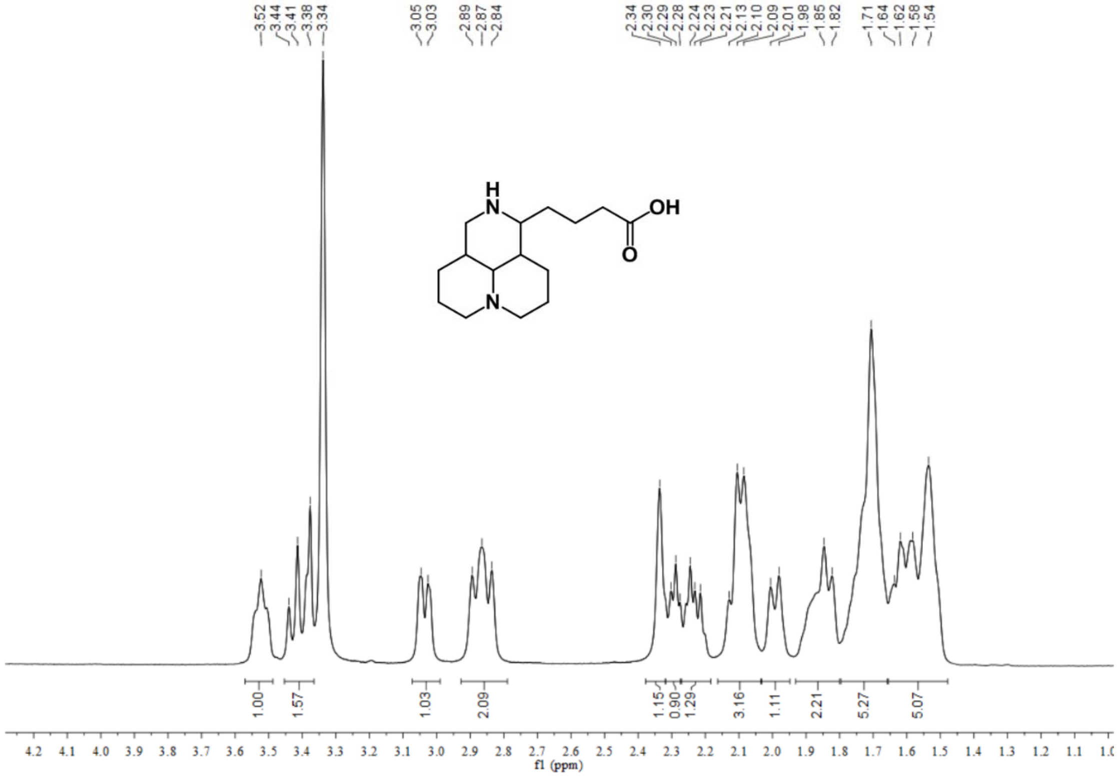

Figure S2. ^{13}C-NMR of **2** in D_2O and CD_3OD.

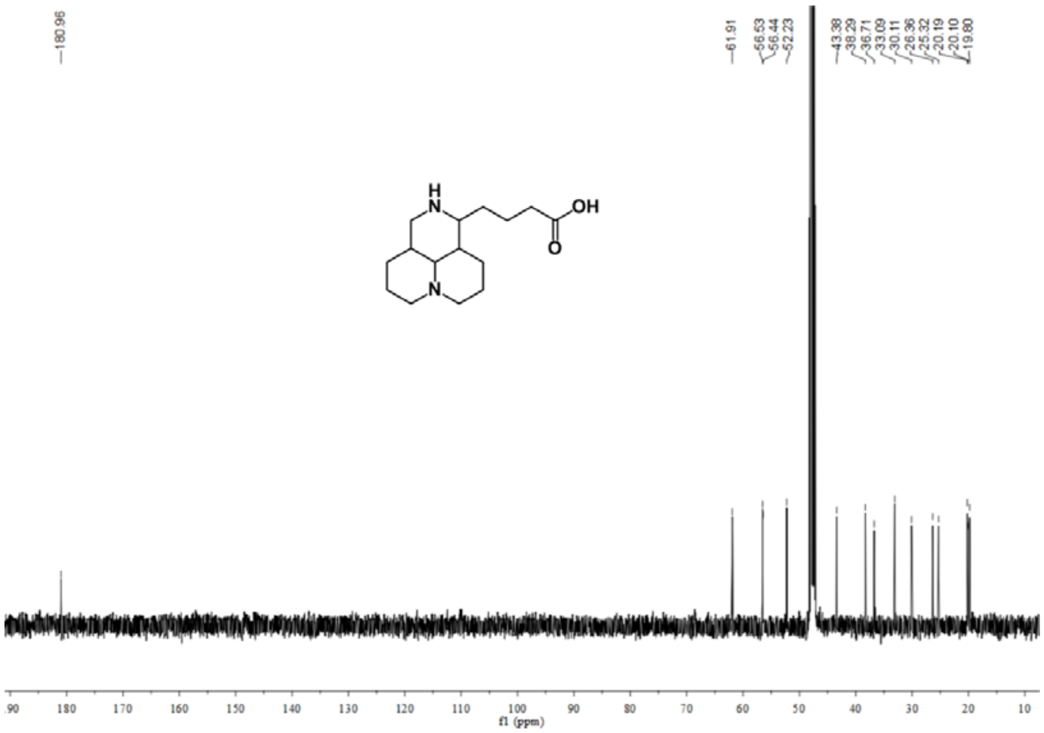

Figure S3. ^{1}H-NMR of **3** in CD_3Cl.

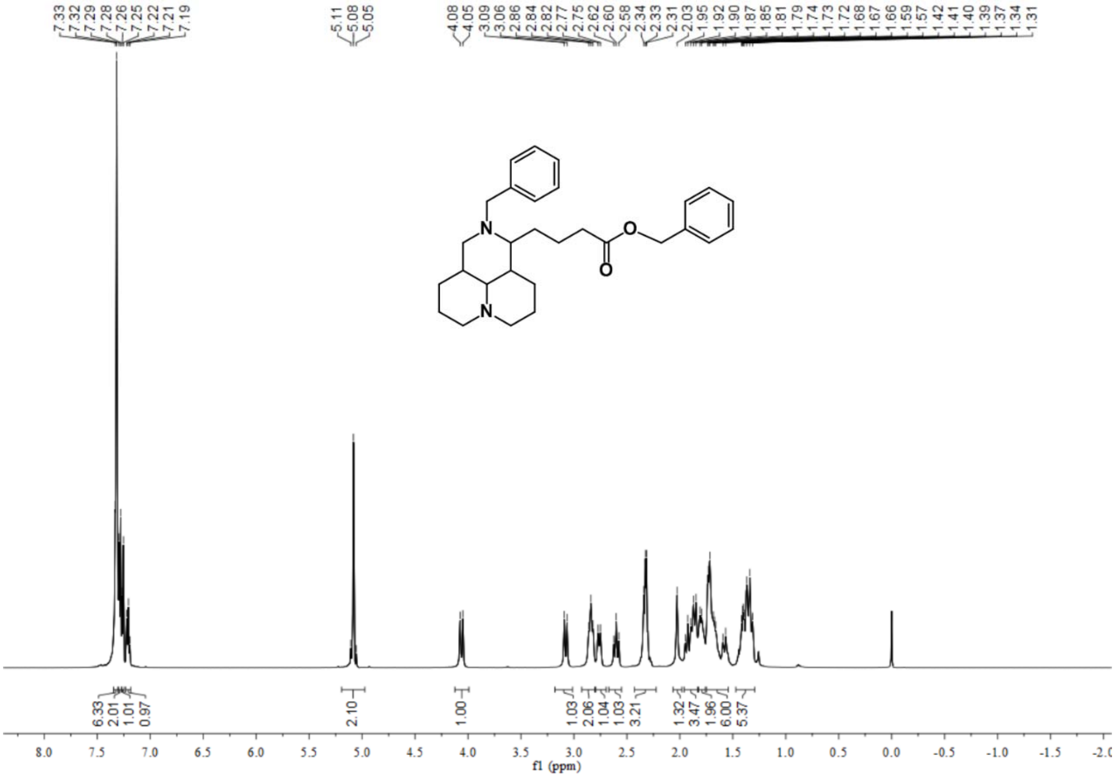

Figure S4. ^{13}C-NMR of **3** in CD_3Cl.

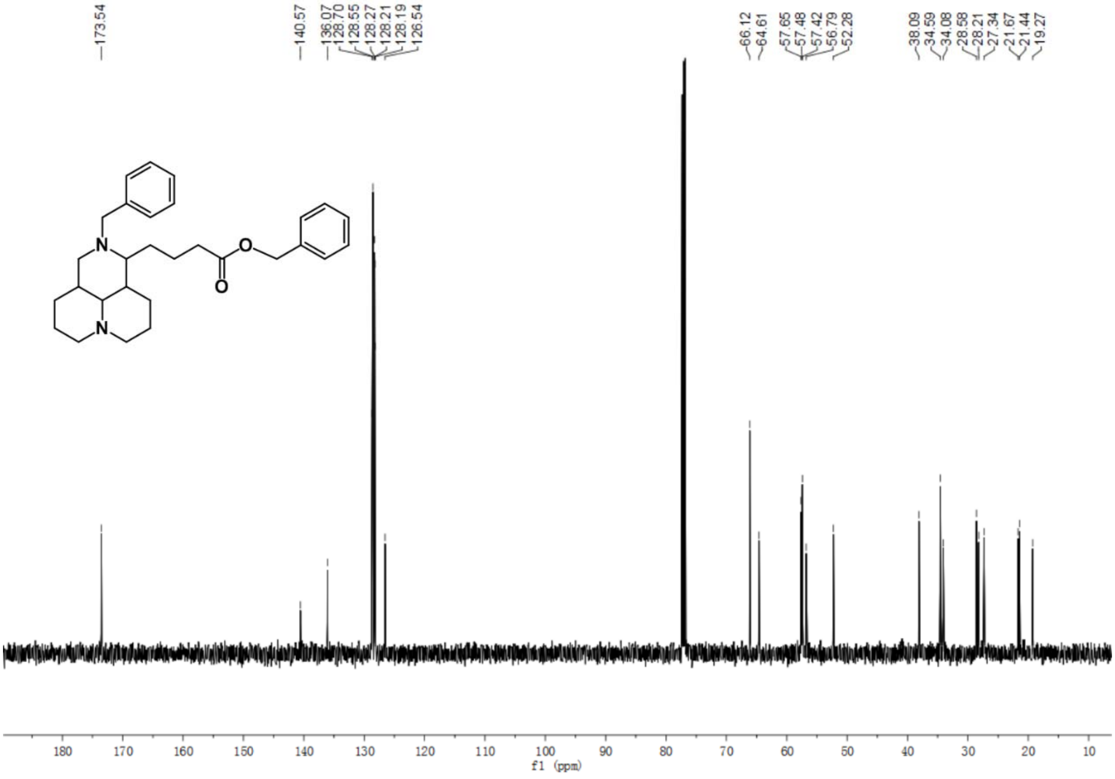

Figure S5. ^{1}H-NMR of **4** in $CDCl_3$.

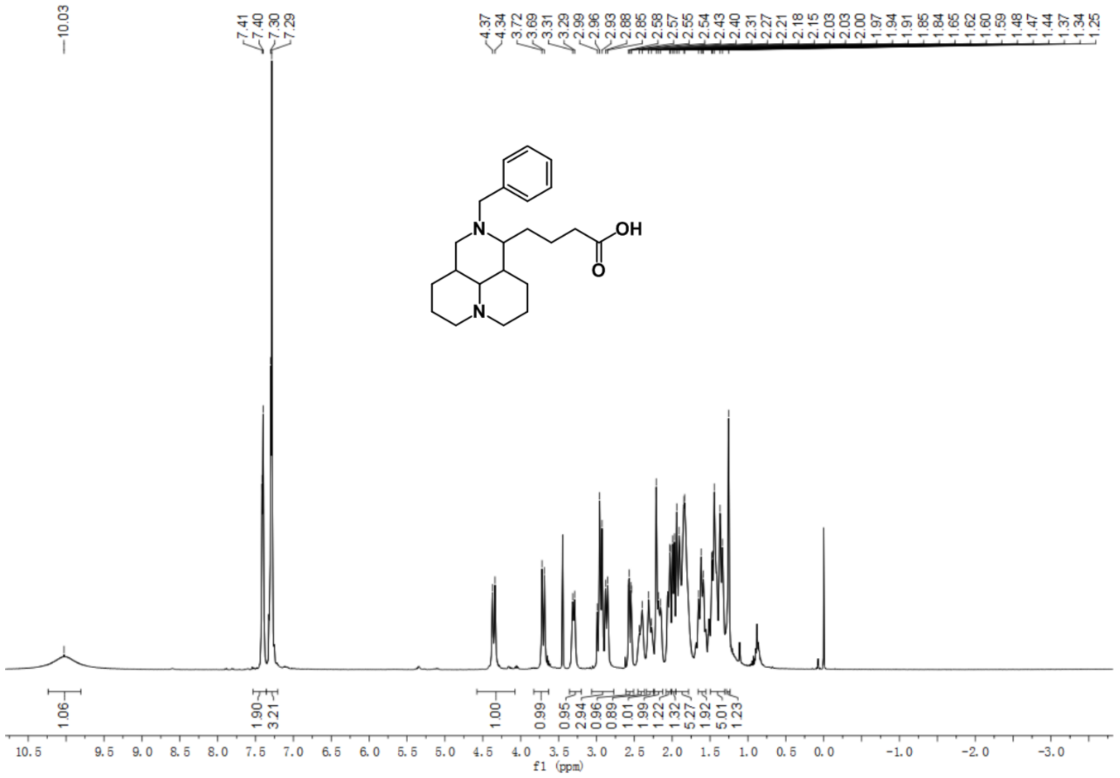

Figure S6. ^{13}C-NMR of **5a** in $CDCl_3$.

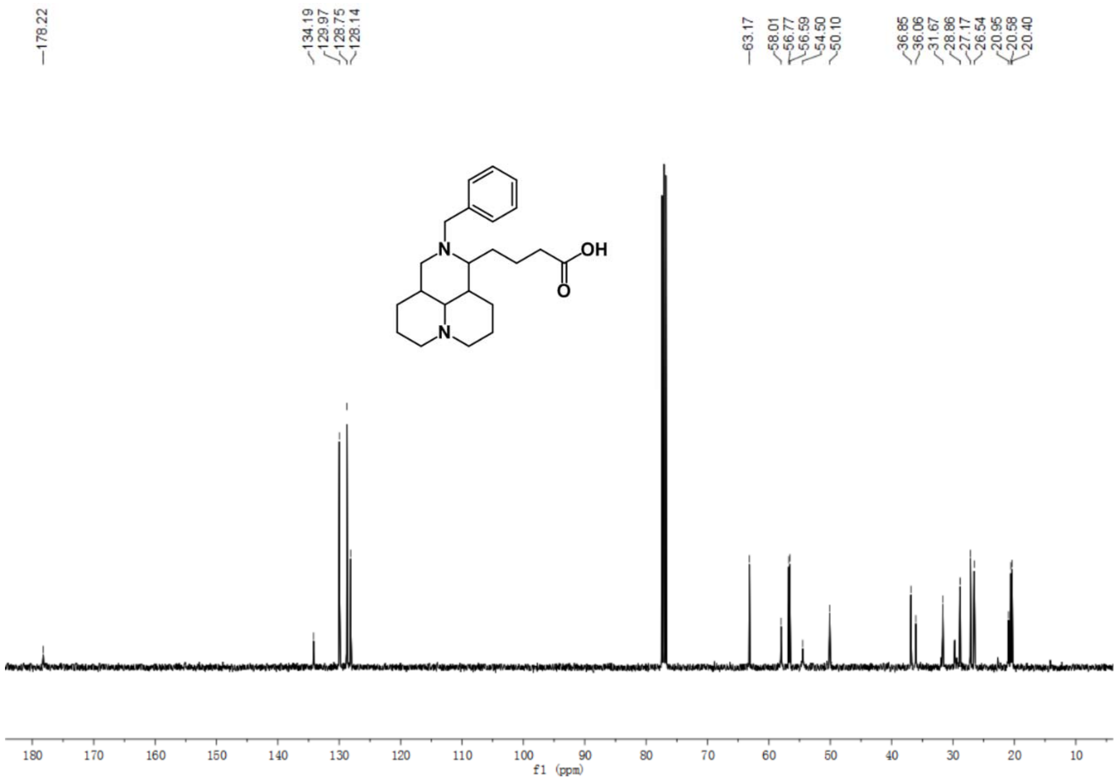

Figure S7. ^{1}H-NMR of **5a** in $CDCl_3$.

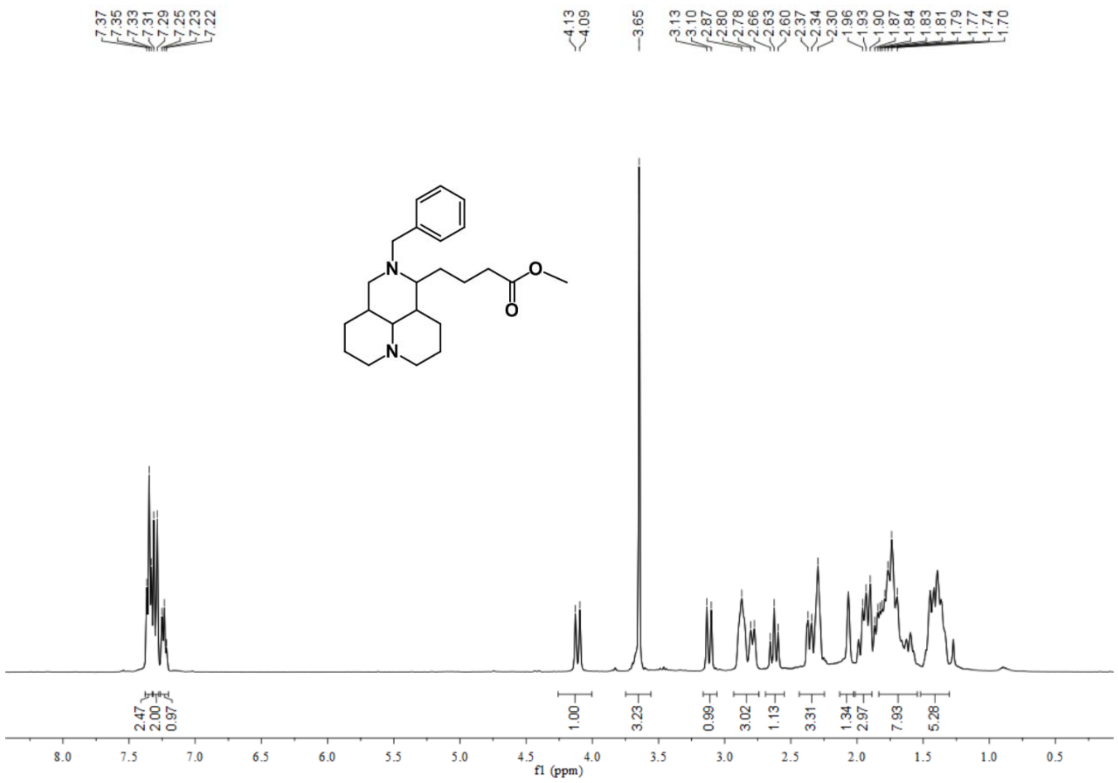

Figure S8. ^{13}C-NMR of **5a** in $CDCl_3$.

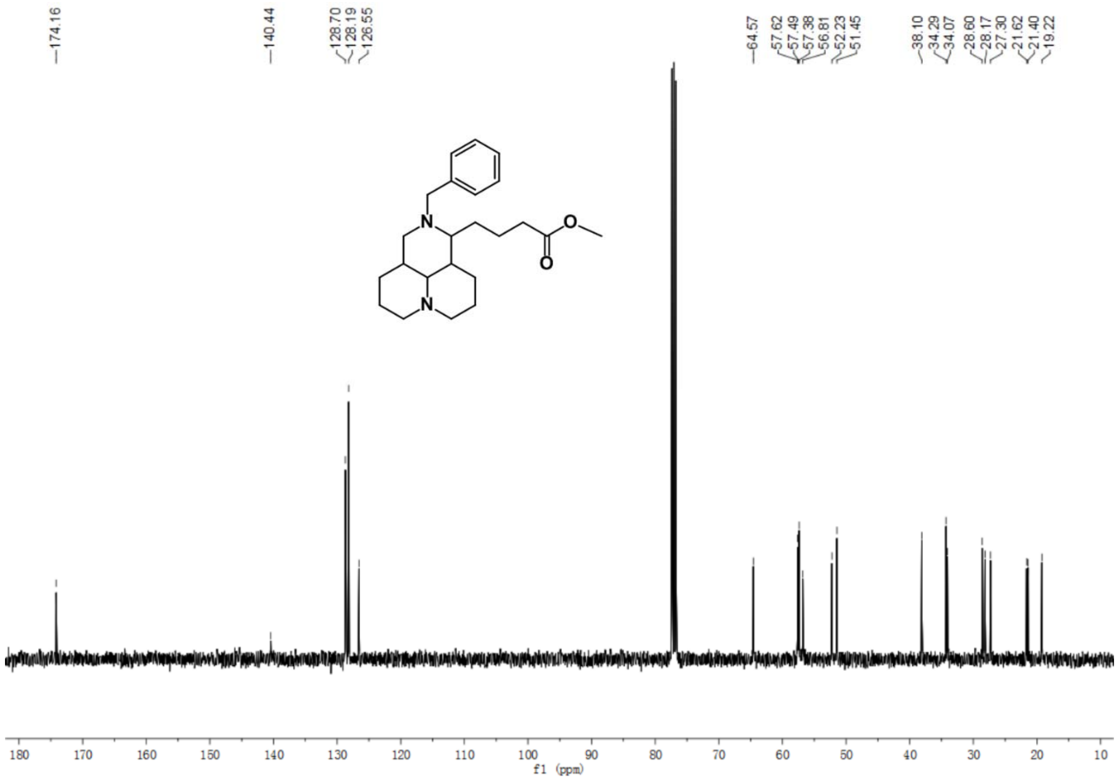

Figure S9. ^{1}H-NMR of **5b** in $CDCl_3$.

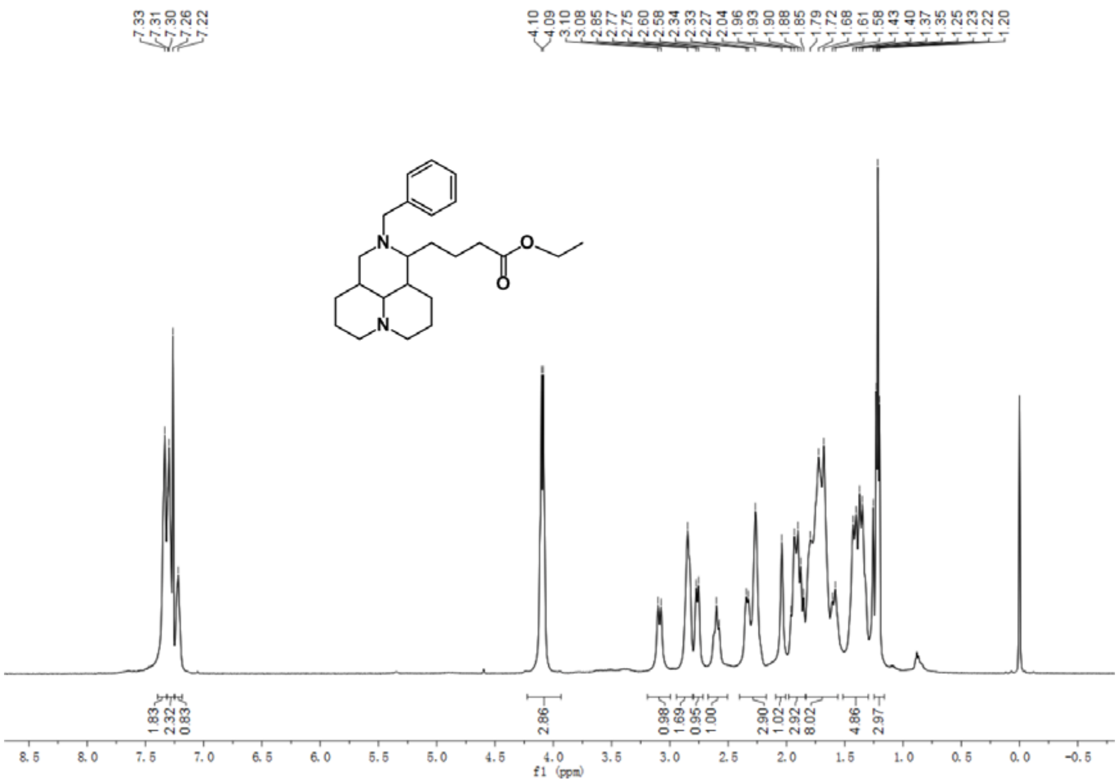

Figure S10. ^{13}C-NMR of **5b** in $CDCl_3$.

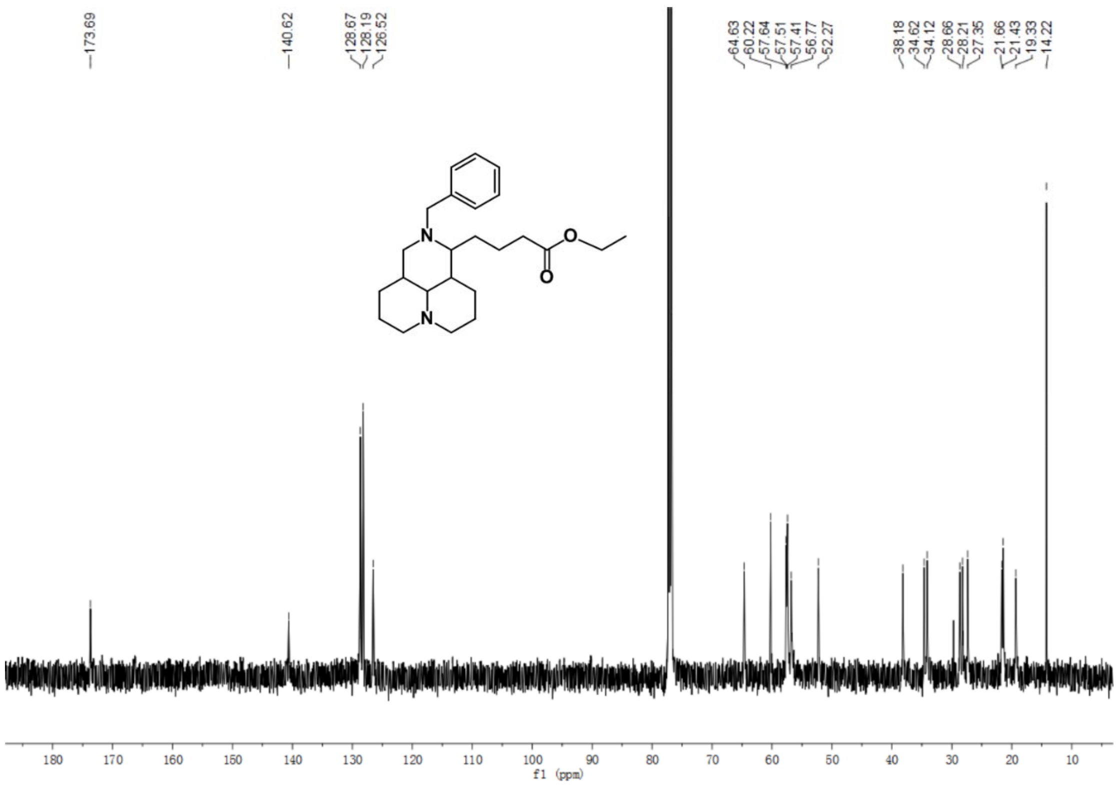

Figure S11. ^{1}H-NMR of **5c** in $CDCl_3$.

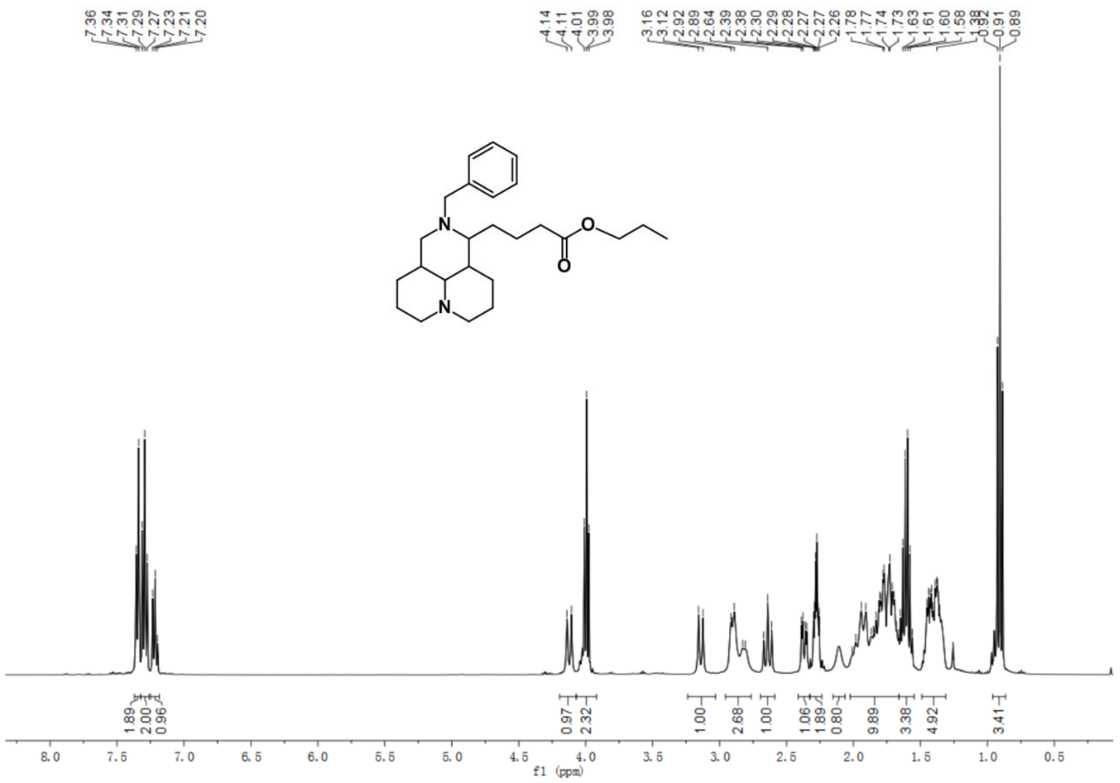

Figure S12. ^{13}C-NMR of **5c** in $CDCl_3$.

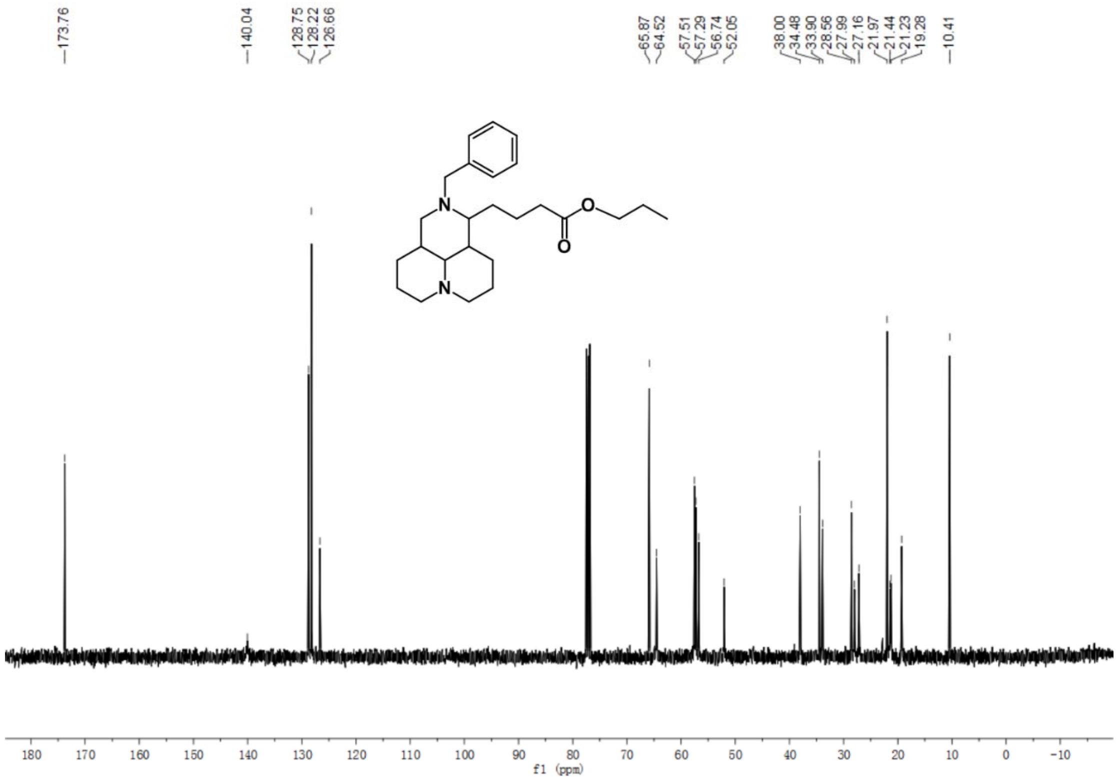

Figure S13. ^{1}H-NMR of **5d** in $CDCl_3$.

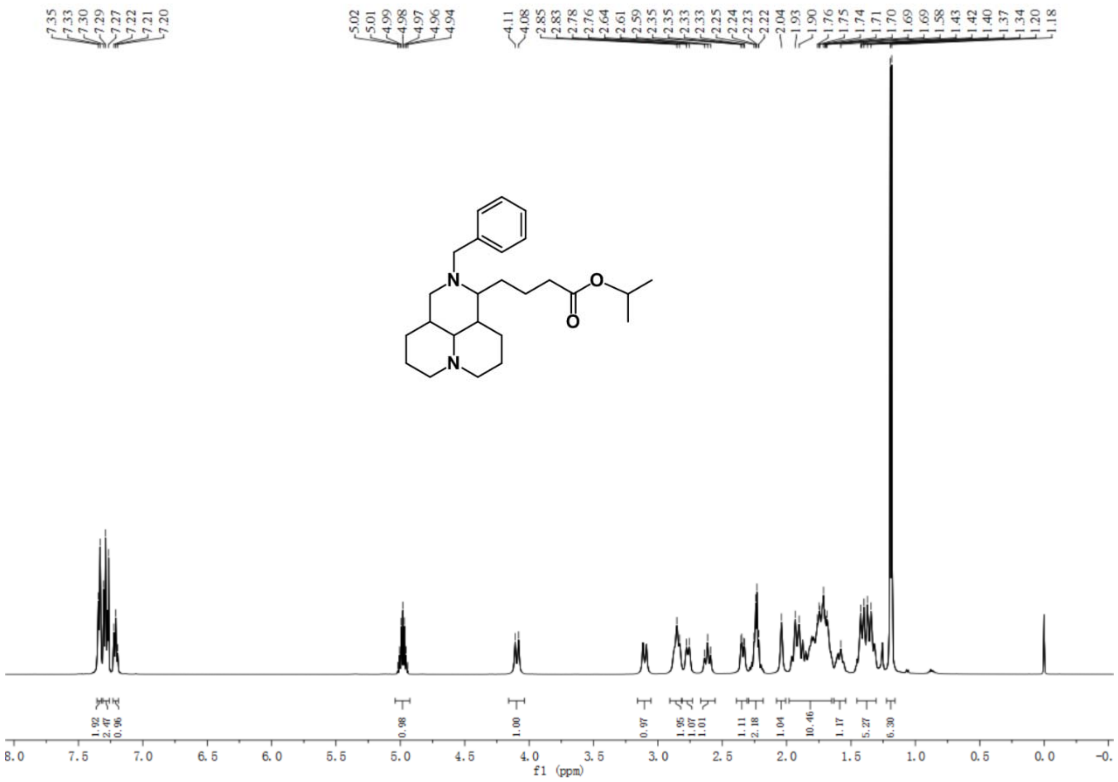

Figure S14. ^{13}C-NMR of **5d** in $CDCl_3$.

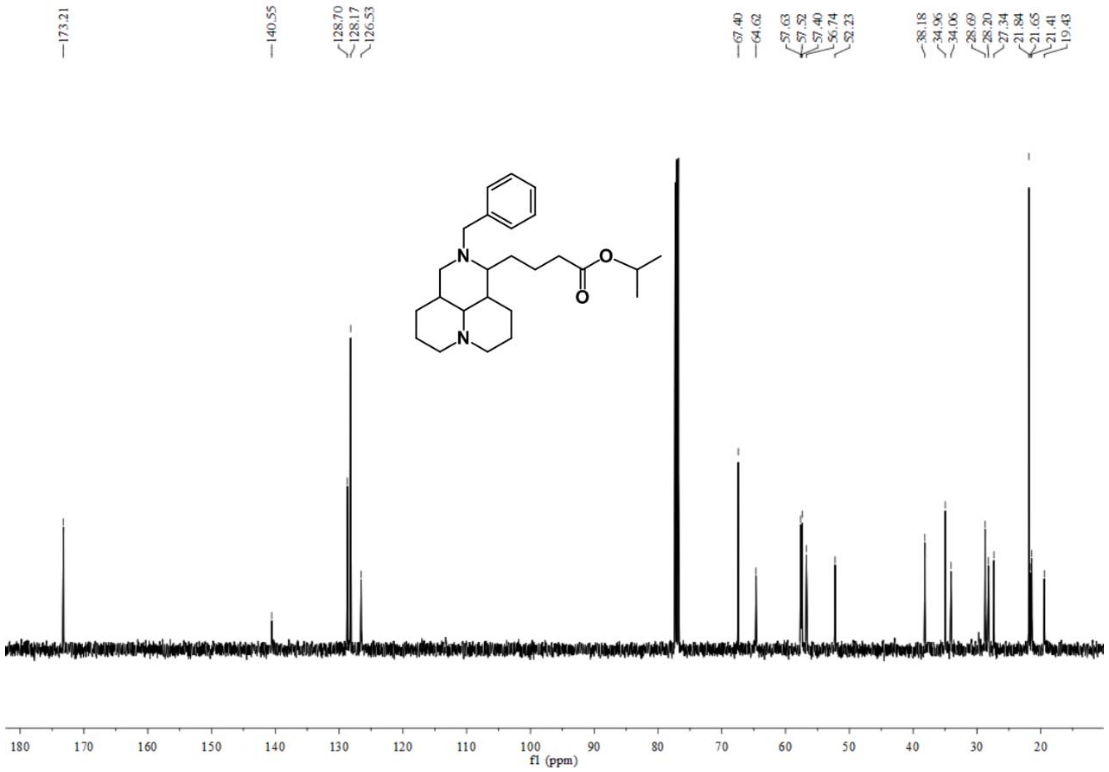

Figure S15. ^{1}H-NMR of **5e** in $CDCl_3$.

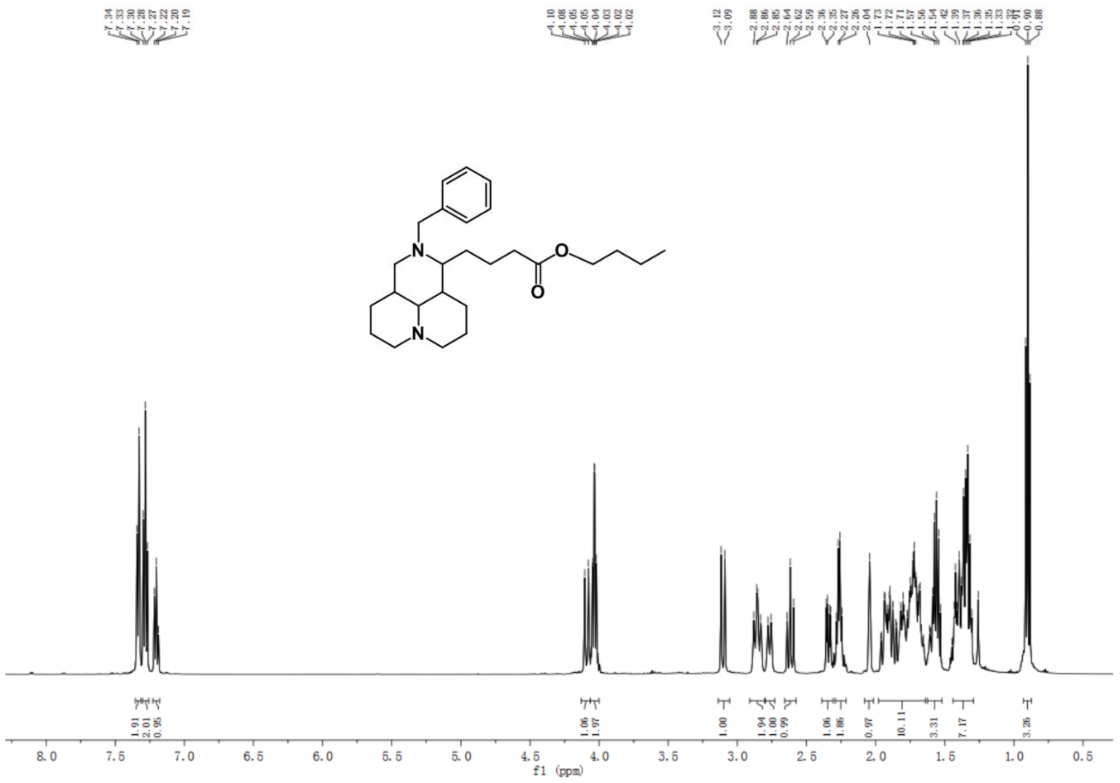

Figure S16. ^{13}C-NMR of **5e** in $CDCl_3$.

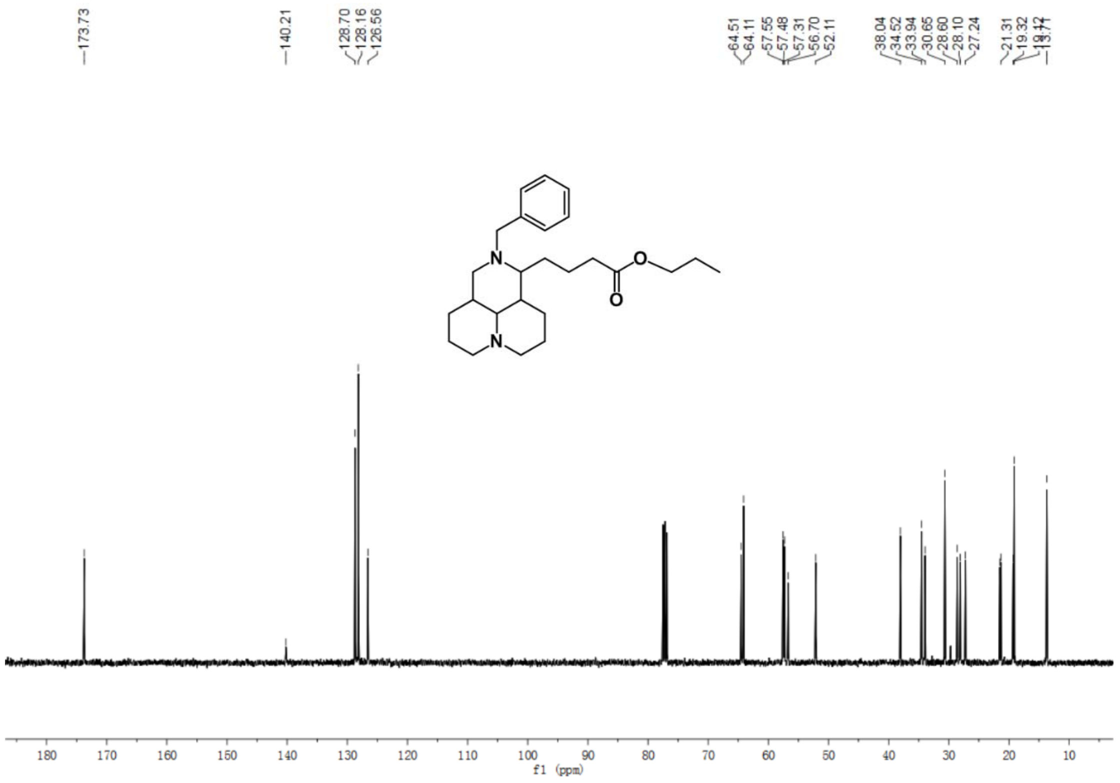

Figure S17. ^{1}H-NMR of **5f** in $CDCl_3$.

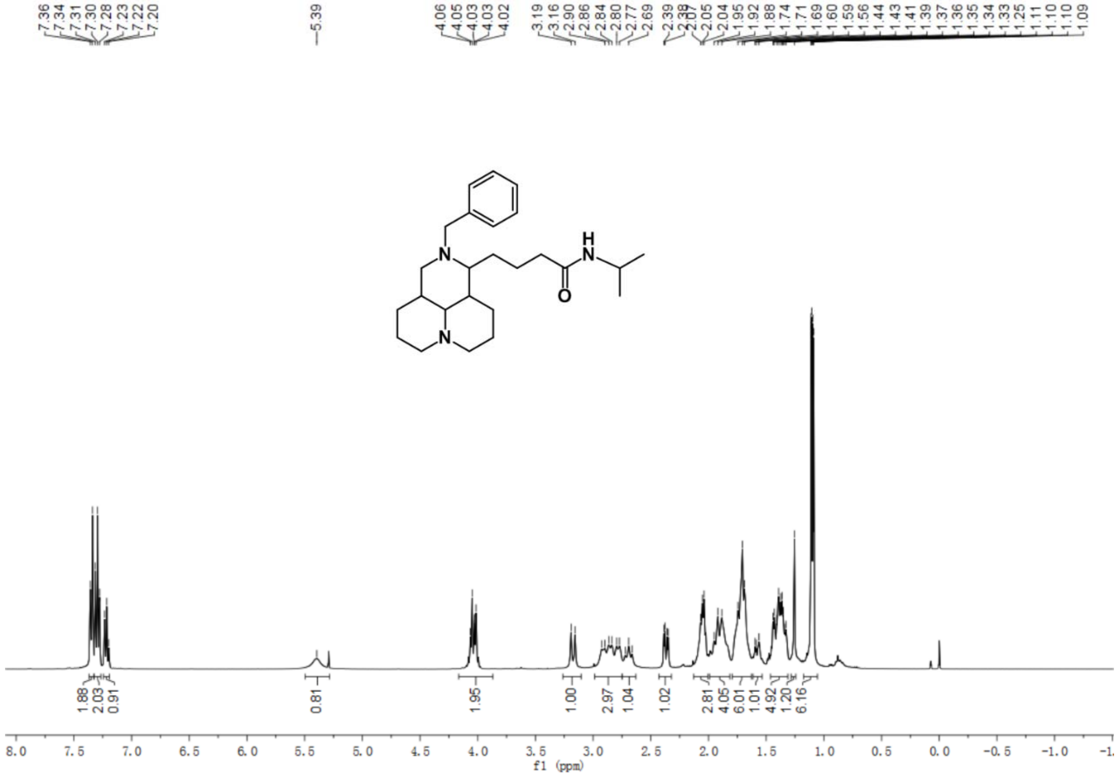

Figure S18. ^{13}C-NMR of **5f** in $CDCl_3$.

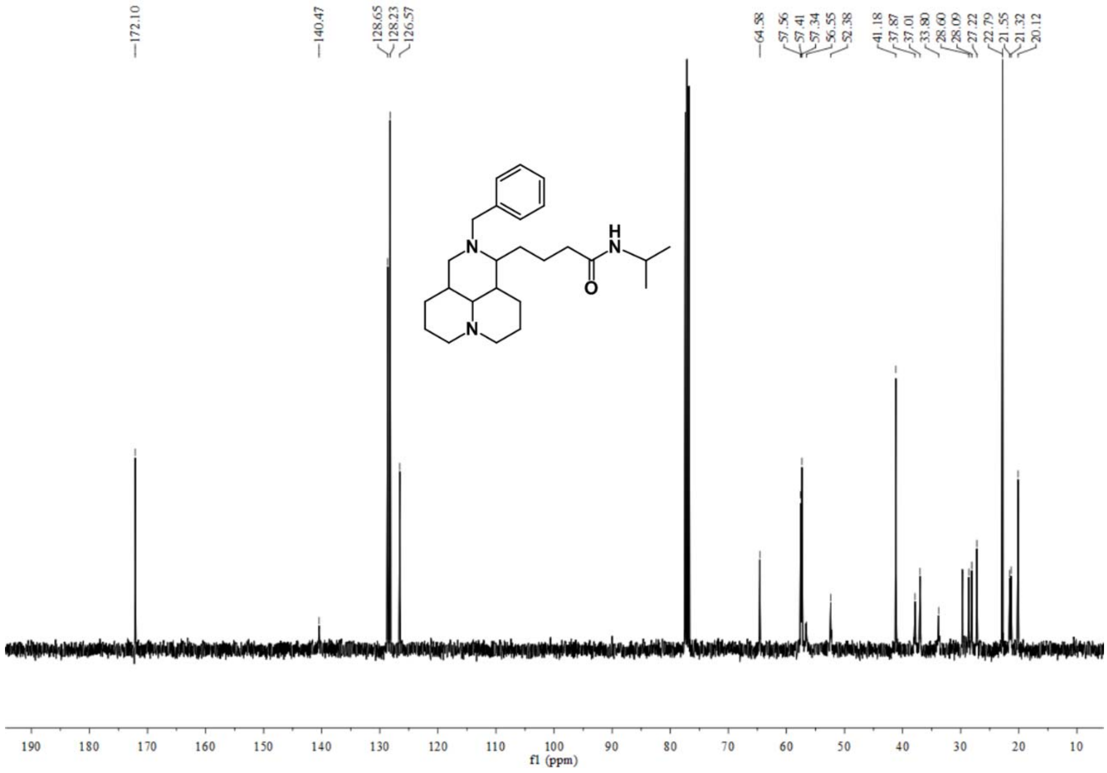

Figure S19. ^{1}H-NMR of **5g** in $CDCl_3$.

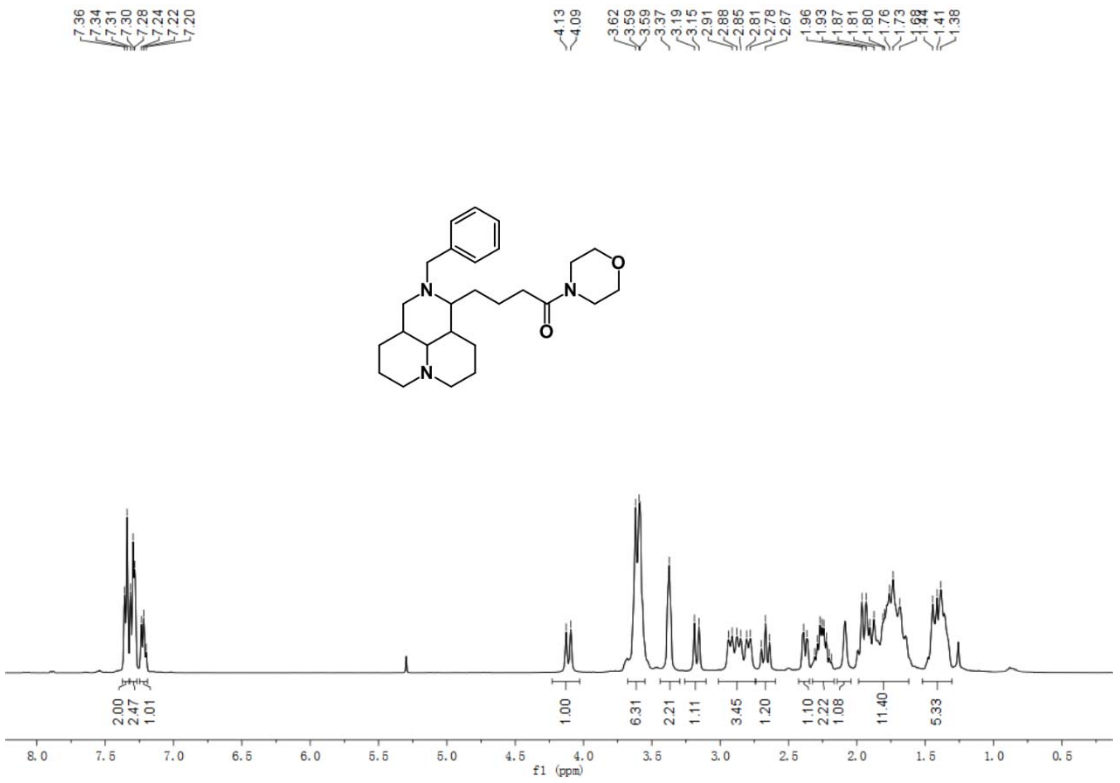

Figure S20. ^{13}C-NMR of **5g** in $CDCl_3$.

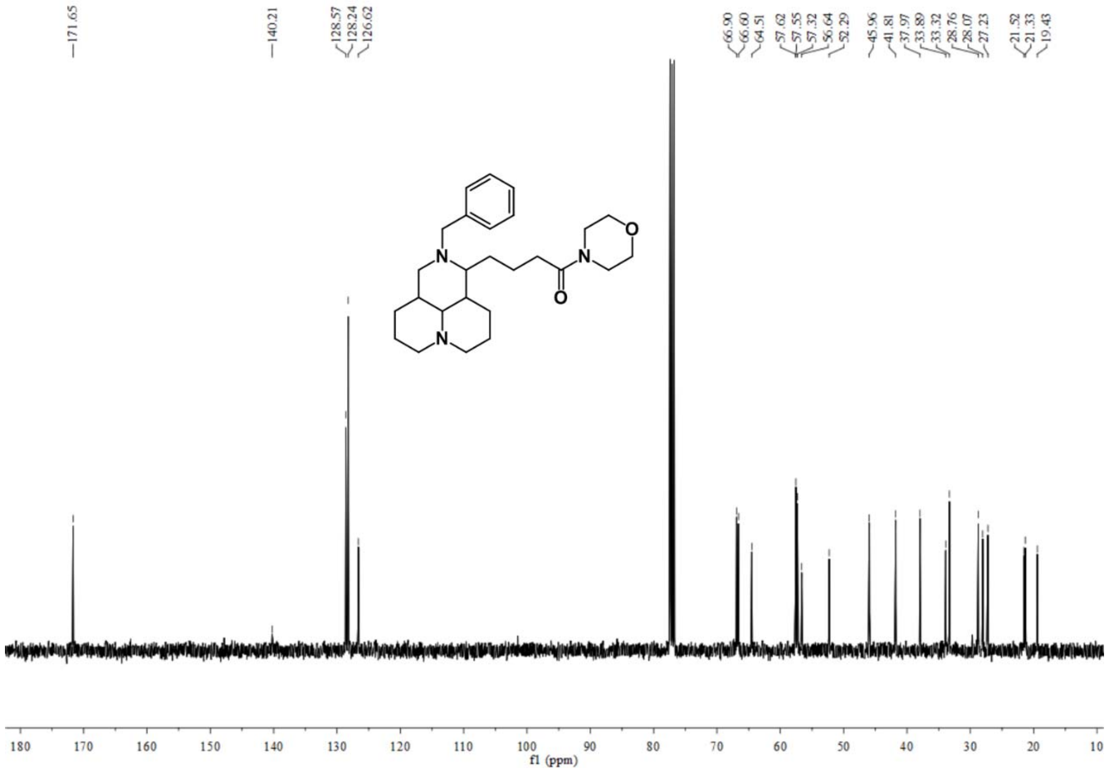

Figure S21. ^{1}H-NMR of **5h** in $CDCl_3$.

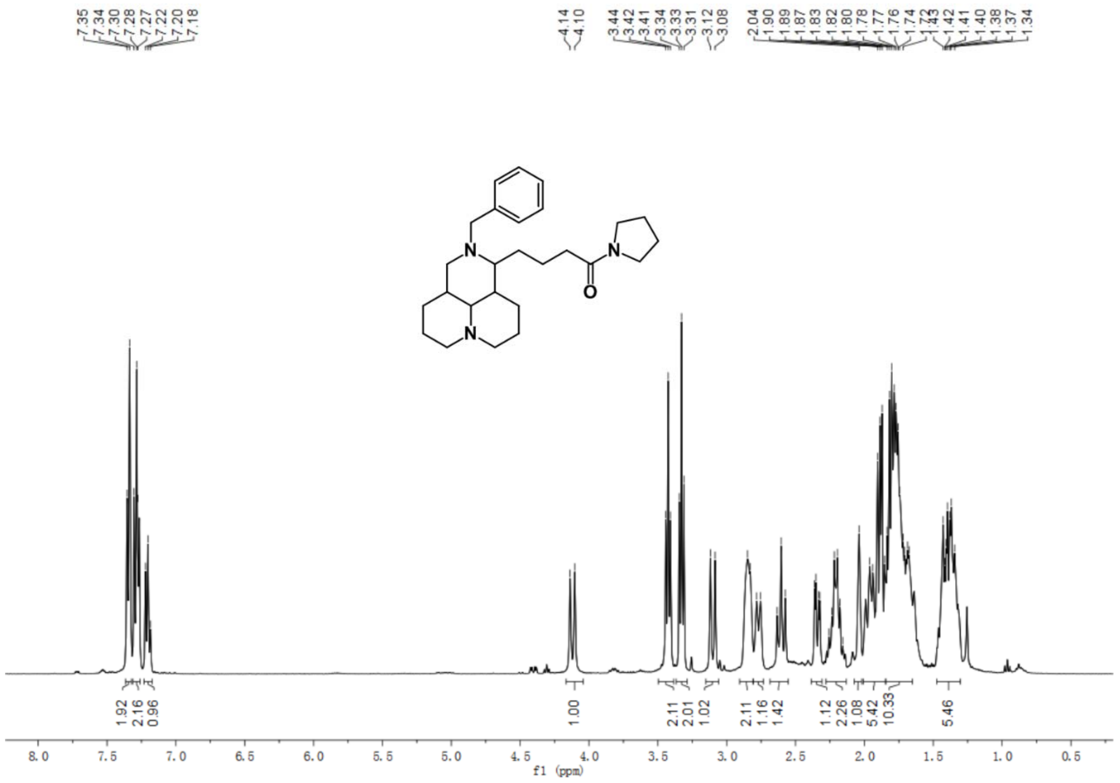

Figure S22. ^{13}C-NMR of **5h** in $CDCl_3$.

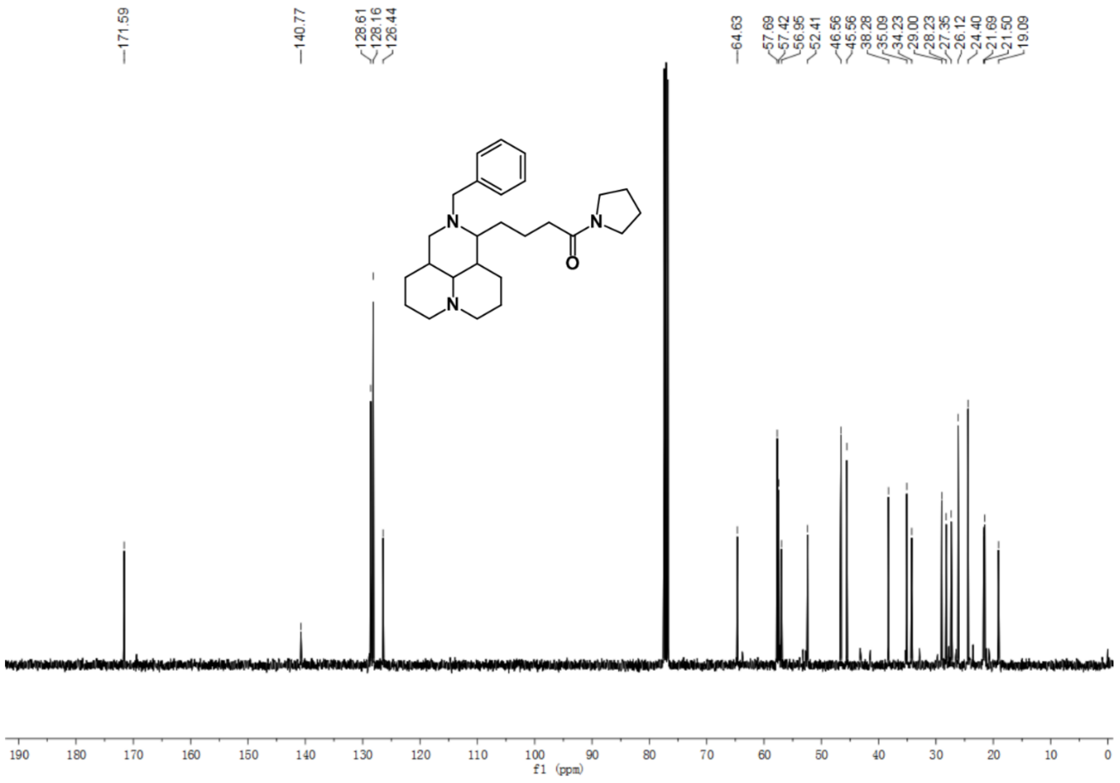

Figure S23. ^{1}H-NMR of **5i** in $CDCl_3$.

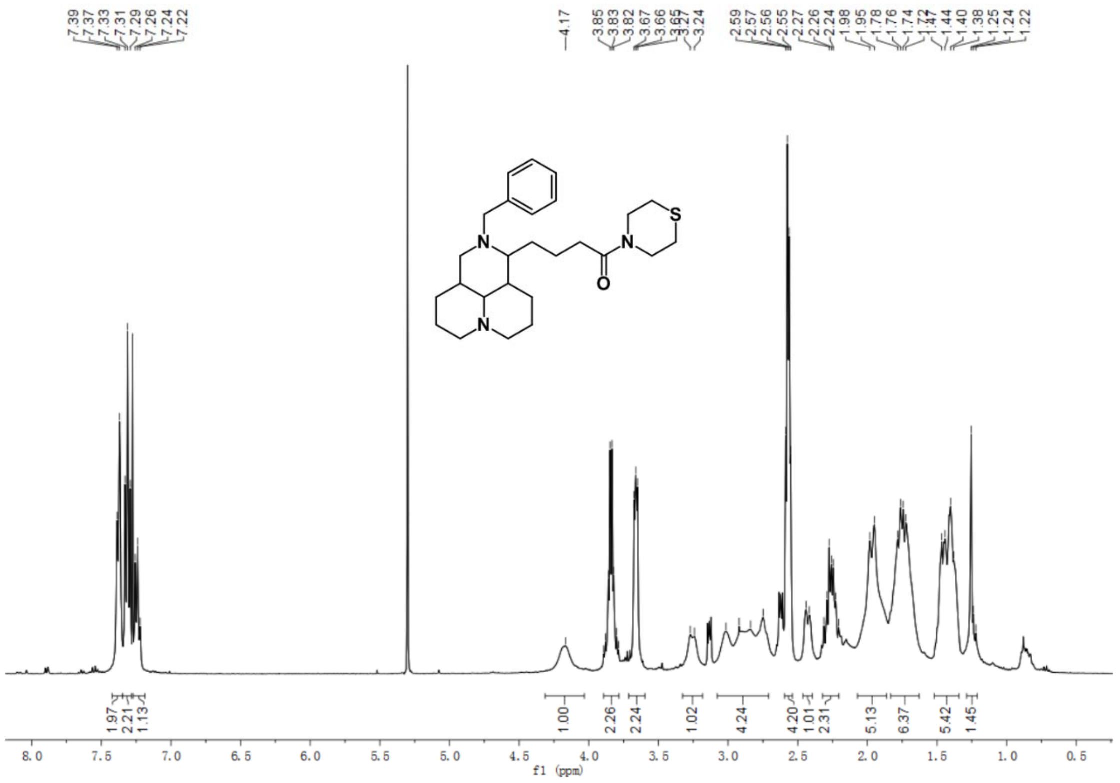

Figure S24. ^{13}C-NMR of **5i** in $CDCl_3$.

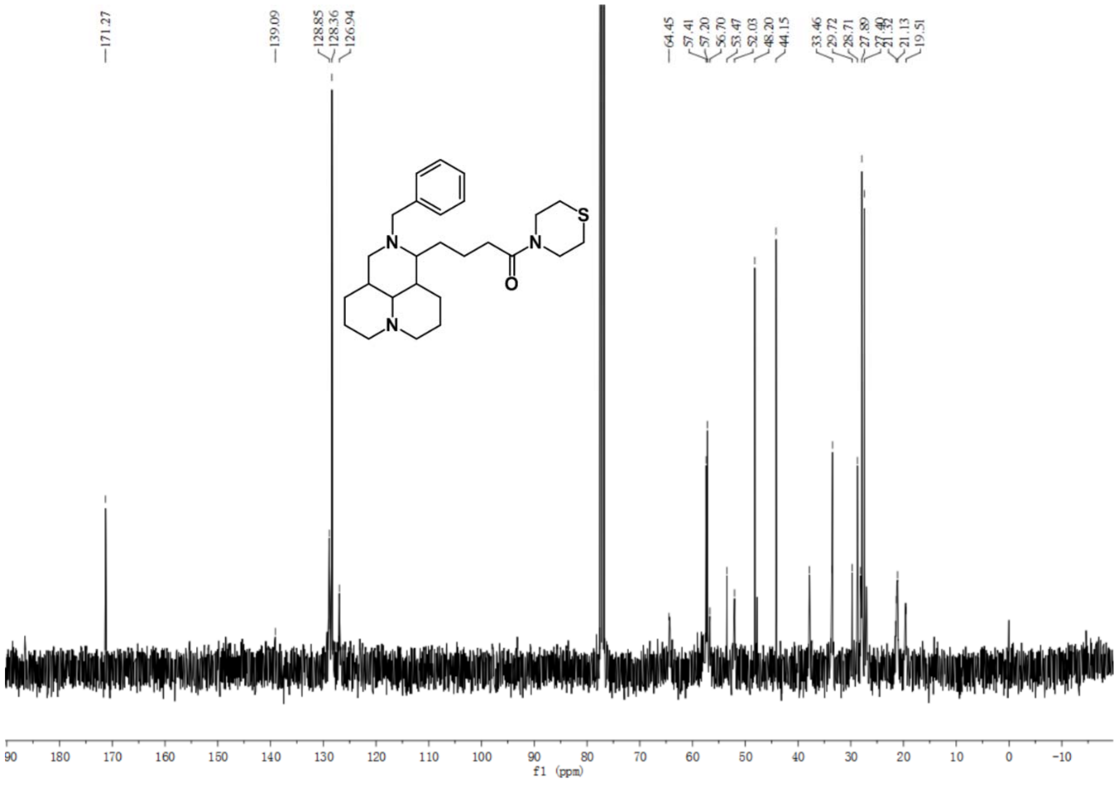

Supplement: Supplementary file 1 [file molecules-18-05420-s001.pdf]
